# Targeted RNA condensation in living cells via genetically encodable triplet repeat tags

**DOI:** 10.1093/nar/gkad621

**Published:** 2023-07-24

**Authors:** Zhaolin Xue, Kewei Ren, Rigumula Wu, Zhining Sun, Ru Zheng, Qian Tian, Ahsan Ausaf Ali, Lan Mi, Mingxu You

**Affiliations:** Department of Chemistry, University of Massachusetts, Amherst, MA 01003, USA; Department of Chemistry, University of Massachusetts, Amherst, MA 01003, USA; School of Chemistry and Chemical Engineering, Nanjing University of Science and Technology, Nanjing 210094, China; Department of Chemistry, University of Massachusetts, Amherst, MA 01003, USA; Department of Chemistry, University of Massachusetts, Amherst, MA 01003, USA; Department of Chemistry, University of Massachusetts, Amherst, MA 01003, USA; Department of Chemistry, University of Massachusetts, Amherst, MA 01003, USA; Department of Chemistry, University of Massachusetts, Amherst, MA 01003, USA; Department of Chemistry, University of Massachusetts, Amherst, MA 01003, USA; Department of Chemistry, University of Massachusetts, Amherst, MA 01003, USA; Molecular and Cellular Biology Program, University of Massachusetts, Amherst, MA 01003, USA

## Abstract

Living systems contain various membraneless organelles that segregate proteins and RNAs via liquid–liquid phase separation. Inspired by nature, many protein-based synthetic compartments have been engineered *in vitro* and in living cells. Here, we introduce a genetically encoded CAG-repeat RNA tag to reprogram cellular condensate formation and recruit various non-phase-transition RNAs for cellular modulation. With the help of fluorogenic RNA aptamers, we have systematically studied the formation dynamics, spatial distributions, sizes and densities of these cellular RNA condensates. The *cis-* and *trans*-regulation functions of these CAG-repeat tags in cellular RNA localization, life time, RNA–protein interactions and gene expression have also been investigated. Considering the importance of RNA condensation in health and disease, we expect that these genetically encodable modular and self-assembled tags can be widely used for chemical biology and synthetic biology studies.

## Graphical Abstract

**Graphical Abstract:**
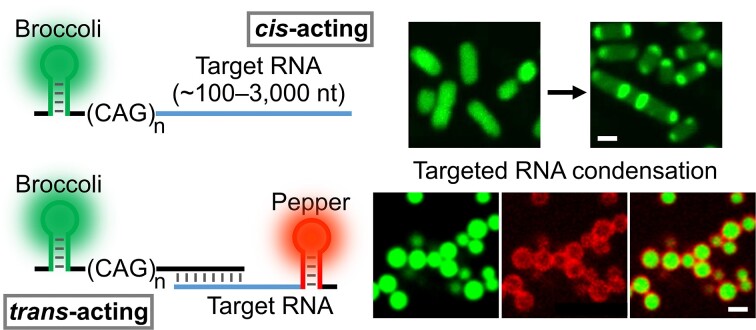
The development of a nature-inspired self-assembled RNA nanodevice that can programmably recruit different RNAs of interest into cellular condensates for the regulation of RNA localization, compartmentalization and functions.

## INTRODUCTION

The functions of cellular RNAs are highly related to their subcellular localizations and local environment. One ubiquitous approach to control RNA localization is via macromolecular condensation, i.e. the formation of membraneless subcellular compartments. RNA compartmentalization is prevalent and plays critical roles in processes such as transcription, splicing, RNA degradation, heterochromatin formation and stress response (1–4). The precise modulation of subcellular compartmentalization of specific RNA sequences is thus important for controlling gene expression and cellular functions.

Cellular RNA localization, compartmentalization and trafficking have been largely regulated by the formation of ribonucleoprotein (RNP) complexes via RNA–protein interactions (5,6). Recent studies demonstrated that specific RNA self-assemblies, particularly among repeat expansions, can also be used to mediate RNA phase separation inside living cells (7–10). Compared with protein-based RNA compartmentalization, RNA–RNA interaction-mediated condensate formation can be highly sequence specific, modular and programmable (11,12). As a result, powerful RNA devices may be engineered to control cellular compartmentalization of specific cellular RNAs and to modulate their local concentrations and functions.

In this study, short CAG trinucleotide repeats are engineered into genetically encodable self-regulated tags to recruit and condense different cellular target RNAs. CAG repeats are used here because these trinucleotides can readily form condensates, without the involvement of proteins (9,10,13,14). We want to test here if these naturally occurring CAG repeats can be used as functional RNA nanodevices, being either a *cis-*acting RNA element within the target RNA transcript or a *trans*-acting effector functioning through specific hybridization with target RNAs. Both *cis-* and *trans*-mechanisms can be potentially applied to develop general platforms to induce the phase separation of various target RNAs, *in vitro* and in living systems.

To image these RNA condensates, especially inside living cells, we fuse the CAG-repeat-tagged RNA strands with a fluorogenic RNA aptamer, named Broccoli. Broccoli is an RNA strand that can selectively bind and activate the fluorescence signal of small molecule dyes, such as DFHBI-1T (15). The dynamics, sizes and densities of RNA condensates can thus be visualized and quantified based on fluorescence images. Our results show that target RNAs of different lengths and sequences can be efficiently recruited into condensates by these CAG-repeat tags. CAG repeats can also be genetically encoded to regulate the subcellular localization, compartmentalization and function of various mRNAs and non-coding RNAs inside living bacterial cells.

## MATERIALS AND METHODS

### Reagents, apparatus and RNA sequences

The reagents, apparatus and RNA sequences used in this study are listed in the Supplementary Data.

### Vector construction

Different lengths of the CAG repeats were first cloned into a pAV-U6 + 27-F30-2×dBroccoli vector. In more detail, the vector was first digested with XbaI and SacII restriction enzymes (NEB). After purification via a 1% agarose gel, the digested vector was ligated together with a similarly digested CAG repeat insert using T4 DNA ligase (NEB). The ligated product was then transformed into *Escherichia coli* BL21 Star™ (DE3) cells (Thermo Fisher Scientific) and selected based on ampicillin resistance.

To construct CAG repeat-expressing pET-28c vectors for bacterial imaging, a pET-28c-F30-2×dBroccoli vector was digested with BgIII and XhoI restriction enzymes (NEB), and then ligated together with a similarly digested CAG repeat insert using T4 DNA ligase. The CAG repeat inserts were synthesized by polymerase chain reaction (PCR) using the above-prepared pAV-U6 + 27-F30-2×dBroccoli-CAG repeat vectors as the template, primers containing the T7 terminator, and BgIII and XhoI restriction sites. After T4 DNA ligation, the product was transformed into BL21 Star™ (DE3) cells and selected based on kanamycin resistance.

The OxyS, *lacY* and *lacZ* fragments were each cloned into pET-28c-F30-2×dBroccoli-CAG repeat vectors after digestion via BsaI and EcoRI restriction enzymes (NEB). The *lacY* and *lacZ* fragments were synthesized by PCR using the pYFP-lacY-1 and pSFV3-lacZ plasmids as the templates, and primers that introduce BsaI and EcoRI restriction sites. After T4 DNA ligation, the product was transformed into BL21 Star™ (DE3) cells and selected based on kanamycin resistance. All these above-prepared plasmids were isolated using a GeneJET Plasmid Miniprep Kit (Thermo Fisher Scientific) and confirmed by Sanger sequencing (Eurofins Genomics).

### *In vitro* RNA transcription and condensate formation

For *in vitro* experiments, all the RNAs were transcribed using a HiScribe™ T7 high-yield RNA synthesis kit (NEB) and purified with G-25 columns. Template DNAs were prepared by PCR amplification from the above-mentioned pAV-U6 + 27- or pET-28c-based F30-2×dBroccoli-CAG repeat vectors. The *in vitro* formation of RNA condensates was prepared by first mixing 4 μM RNAs in buffer containing 10 mM Tris–HCl at pH 7.5, 100 mM KCl and 20 mM MgCl_2_. The mixture was then heated up to 95°C for 3 min and cooled down to 37°C at a rate of 2°C/min in a thermocycler. Afterwards, 80 μM DFHBI-1T was added and incubated for 15 min at 37°C before imaging using a Yokogawa spinning disk confocal on a Nikon Eclipse-TI inverted microscope. Images were collected with an excitation wavelength at 488 nm using a ×100/1.45 NA oil immersion objective. The partition ratio of each RNA condensate was calculated based on the ratio of average fluorescence intensity within the condensate versus that in the solution region free of condensates. For the *in vitro* kinetic measurements, a solution containing 10 mM Tris–HCl at pH 7.5, 100 mM KCl, 20 mM MgCl_2_, 4 μM RNA and 80 μM DFHBI-1T was first heated up at 95°C for 3 min and then rapidly cooled down in ice for 30 s immediately before starting to collect images.

### Cellular imaging

RNA imaging in living bacterial cells was performed according to a previously established protocol (16). Briefly, the BL21 Star™ (DE3) cells or *Pseudomonas aeruginosa* cells that express the corresponding RNAs were first grown in LB medium at 37°C until the optical density at 600 nm (OD_600_) reaches 0.4, and then 1 mM isopropyl-β-d-thiogalactopyranoside (IPTG) was added for a 2 h induction. After the IPTG induction, the cells were adhered to a poly-l-lysine-pre-treated glass-bottom 8-well imaging plate (Cellvis, Mountain View, CA, USA) in Dulbecco’s phosphate-buffered saline (DPBS) buffer for 45 min. Then the buffer was switched to fresh DPBS containing 200 μM DFHBI-1T and/or 1 μM HBC620 for a 30 min incubation at 25°C before imaging. All the confocal fluorescence images were collected with NiS-Elements AR software using a Yokogawa spinning disk confocal on a Nikon Eclipse-TI inverted microscope. Broccoli fluorescence signals were excited with a 488 nm laser, Pepper fluorescence signals were excited with a 561 nm laser and the near-infrared fluorescent protein signal was collected under 640 nm laser irradiation. A ×100/1.45 NA oil immersion objective was used for collecting these cellular images. Structured illumination microscopy (SIM) super-resolution imaging was performed on a Nikon A1R-SIMe microscope equipped with a Hamamatsu sCMOS camera and a ×100 oil immersion objective, under 488 nm laser irradiation.

### Fluorescence recovery after photobleaching (FRAP)

The FRAP measurements were performed on an A1 spectral confocal microscope with a ×100 oil immersion objective on a Nikon Eclipse-TI inverted system with an A1 stimulation module to ensure bleaching of a targeted area. Photobleaching was carried out with a 488 nm laser for 1.0 s on a region of ∼1 μm diameter. The fluorescence recovery after bleaching was then imaged every 5 s for a total of 3–5 min.

### Imaging data analysis

Image analysis was performed using NiS-Elements AR Analysis software. The sets of actions performed on imaging channels were built as analysis recipes in the General Analysis 3 (*GA3*) module. For *in vitro* fluorescence images, a fluorescence intensity ‘threshold’ F_B_ + 3SD (background plus 3-fold of standard deviations on the 488 nm channel) and a diameter ‘threshold’ of 0.5 μm was set to automatically detect each condensate. For each identified condensate, the ‘mean object intensity’ and ‘object area’ actions were applied to measure their corresponding mean fluorescence intensity and the area. The number of identified objects in one image (imaging view 4430 μm^2^) equals the measured density of condensates. An inverted threshold was also set for measuring mean background intensities for the calculation of partition ratios.

For bacterial fluorescence images, after applying a ‘smooth’ action, a fluorescence intensity ‘threshold’ F_B_ + 3SD (whole well background plus 3-fold of standard deviations on the 488 nm channel) was set to automatically detect each individual *E. coli* cell. The averaged whole-cell fluorescence was measured via the ‘mean object intensity’ action. To detect intracellular foci, the ‘bright spots’ action was applied based on a fluorescence intensity ‘threshold’ F_B_ + 3SD (cellular background plus 3-fold of standard deviations on the 488 nm channel) and a diameter ‘threshold’ of 0.5 μm. ‘Contrast’ and ‘grow’ were also set to enable the proper detection of foci. Meanwhile, the ‘child ID’ action was applied to set the detected cells as ‘parent’ and the detected foci as ‘child’ and chose ‘child is inside parent’ condition. The count of foci in each detected cell was then obtained using the ‘group records’ and ‘aggregate rows’ actions. The ‘subtract’ action was used to subtract the detected foci region from the detected cells region, i.e. the cellular background region. Then, the ‘mean object intensity’ action was applied on both the foci region and the cellular background region to measure their corresponding mean fluorescence intensities and the partition ratio of each cellular condensate.

To further avoid inappropriate detection of cells and foci, only rod-shaped singly detected cells with properly detected foci were picked and also manually verified. The results shown in this study were generated by at least 90 above-verified cells from at least three representative images unless mentioned otherwise. At least two replicated experiments were performed for all these measurements. All the data analysis and fitting were performed using the ImageJ/FIJI and GraphPad Prism 9.2.0 software. Two-tailed Student's *t*-test was used to determine the statistical significance.

## RESULTS

### *In vitro* RNA condensation

**Figure 1.**
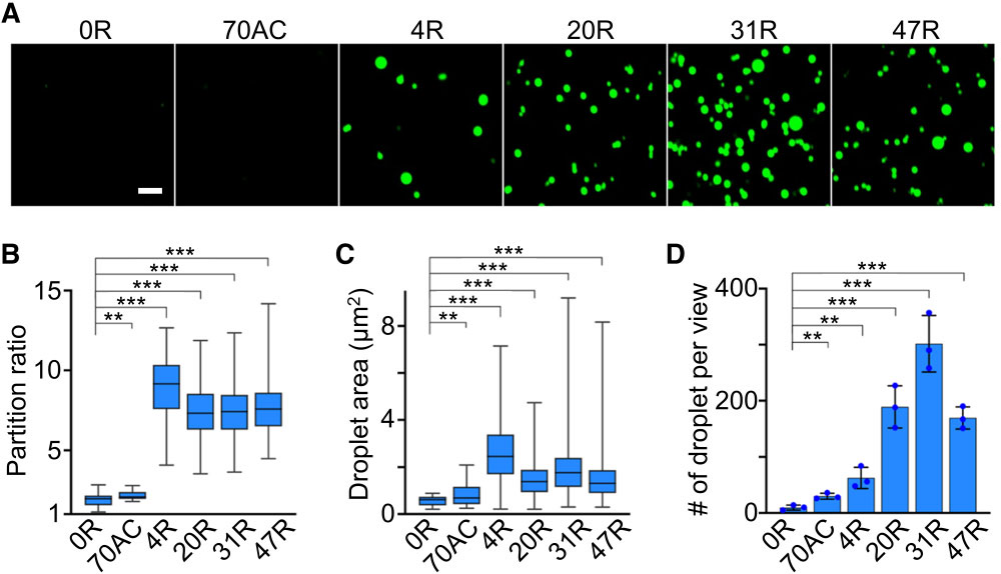
CAG-repeat-regulated *in vitro* RNA condensation. (**A**) Confocal fluorescence imaging of RNA condensates induced by F30-2d×Broccoli-tagged 4×, 20×, 31× or 47× CAG repeats (4R, 20R, 31R or 47R). F30-2d×Broccoli (0R) and F30-2d×Broccoli-tagged 70 repeats of AC (70AC) were used as the negative control. Fluorescence images were taken in solutions containing 4 μM RNA, 20 mM MgCl_2_ and 80 μM DFHBI-1T after annealing. Scale bar, 5 μm. (**B**) The partition ratio, i.e. the ratio of average fluorescence intensity inside individual condensate versus background solution signals, and (**C**) the droplet area of each type of RNA condensate is plotted in a box with minimum to maximum whiskers. The top and bottom line, upper and lower box boundary and inner line indicate the minimum and maximum data point excluding outliers, 75th and 25th percentile and median of the data, respectively. A total of 20, 29, 241, 318, 323 and 437 condensates were analyzed in the case of 0R, 70AC, 4R, 20R, 31R and 47R, respectively. (**D**) The number of droplets observed per imaging view. Each imaging view equals 4430 μm^2^. Shown are the mean and the standard deviation (SD) values from at least three representative images. Two-tailed Student's *t*-test: ***P< 0.001; **P< 0.01.

We first asked if Broccoli can be used to visualize CAG-repeat-induced RNA condensation. To test this, we synthesized F30-2d×Broccoli-tagged RNA strands containing 0×, 4×, 20×, 31× or 47× CAG repeats, which were named 0R, 4R, 20R, 31R and 47R, respectively. The F30 scaffold was used to ensure the proper folding of two incorporated dimeric Broccoli RNAs (Supplementary Table S1) (17). Compared with untagged F30-2d×Broccoli (i.e. 0R), after attaching these RNA repeats, F30-2d×Broccoli still exhibits strong, even slightly higher, fluorescence signals (Supplementary Figure S1A). By annealing a solution containing 4 μM RNA, 20 mM MgCl_2_ and 80 μM DFHBI-1T, the 20R, 31R and 47R samples exhibited obvious RNA condensation (Figure 1A), with a large number of spherical-shaped fluorescent condensates at ∼1.4–1.8 μm in diameter (Figure 1C). In contrast, almost no phase separation was observed with the 0R and 70AC control. Here, 70AC is an F30-2d×Broccoli-tagged RNA of equivalent length to 47R but contains only repeated AC dinucleotides. Interestingly under our experimental condition, with only 4× CAG repeats, 4R can also generate large-sized RNA condensates (diameter, ∼1.8 μm), while at a lower density than longer CAG repeats.

To further compare the RNA densities within these different CAG-repeat aggregates, we characterized the partition ratio of each single condensate, which is defined as the ratio of average fluorescence intensity inside individual condensate versus background solution fluorescence signals. A partition ratio of ∼2.2, 8.9, 7.4, 7.3 and 7.7 was exhibited for the 70AC, 4R, 20R, 31R and 47R condensates, respectively (Figure 1D). 4R exhibited a slightly higher partition ratio and larger aggregation size compared with other expanded CAG repeats. On the other hand, 4R solution contained fewer condensates (∼5 per 400 μm^2^ imaging area) than that of 20R, 31R and 47R (>15 counts per same area) (Figure 1). These results indicated that the CAG repeats can indeed induce *in vitro* RNA phase separation. Meanwhile, Broccoli can be used as a fluorescence imaging reporter for RNA condensates, and these fluorescence signals may also be used to estimate RNA concentrations within each condensate (Supplementary Figure S1B, C).

We noticed that these CAG-repeat condensates tend to exhibit gel-like behaviors, such as stacking and slow fusion (Figure 1A). These gel-like properties can be the result of strong multivalence interactions among these trinucleotide repeats (8,18). A FRAP approach was also applied to measure RNA mobilities in condensates. Minimal fluorescence recovery was observed (over a total of ∼5 min, Supplementary Figure S2), indicating that *in vitro* formed CAG condensates indeed display a highly static structure. The kinetics of RNA condensation were also monitored right after the snap-cooling of a solution containing 4 μM 47R and 20 mM MgCl_2_. Our results showed that the number of RNA condensates kept increasing during the first 15 min of incubation, while the diameter and partition ratio of each condensate already reached ∼90% of the maximum level in ∼5 min (Supplementary Figure S3). All these data indicated the fast assembly kinetics of these RNA condensates.

We also studied the effect of RNA and Mg^2+^ concentrations on the CAG-repeat-induced phase separation. In our test, 0.02–10 μM of the 0R, 20R, 31R or 47R strands were separately mixed with 5–40 mM MgCl_2_. As expected, the formation of RNA condensates can be facilitated with increasing concentrations of RNA and MgCl_2_ (Supplementary Figure S4). RNAs with longer CAG repeats, e.g. 31R and 47R, can more readily form condensates, even at reduced RNA and MgCl_2_ levels. It is worth mentioning that in these tests, almost no RNA condensation was observed under physiologically relevant ≤5 mM Mg^2+^ ion conditions (19,20). Meanwhile, minimal RNA condensation was shown without annealing. However, intracellular RNA compartmentalization can still be quite different from these *in vitro* tests, as RNA condensation can be potentially facilitated by the crowded and protein-rich cellular environment (7,21,22).

### CAG-repeat-mediated target RNA condensation

**Figure 2.**
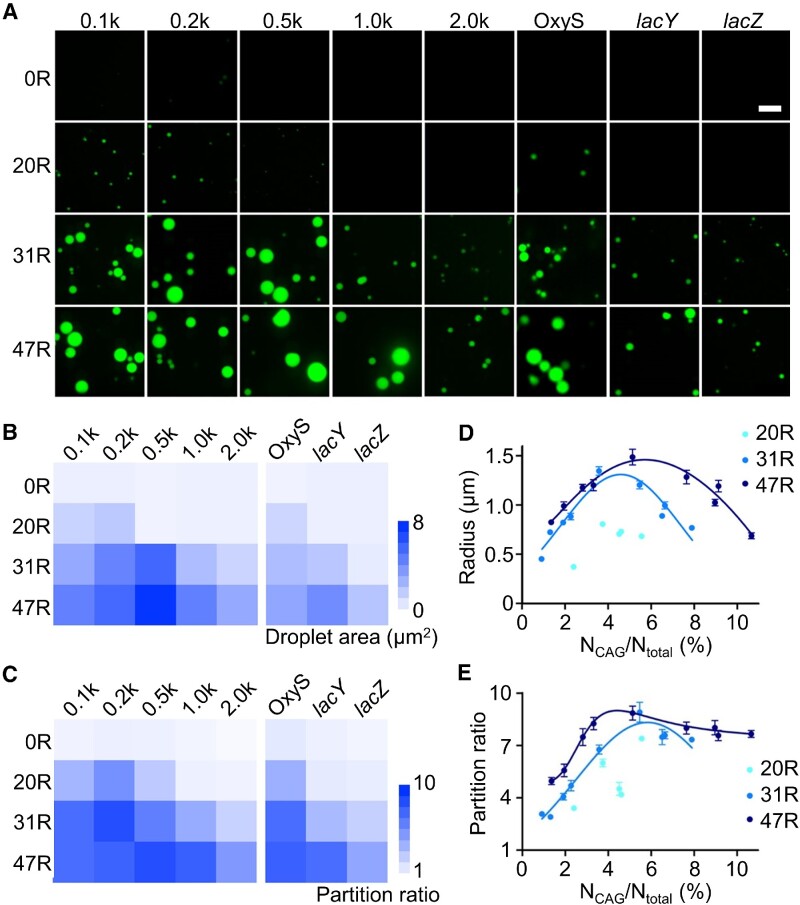
Targeted RNA condensation via CAG repeats. (**A**) Confocal fluorescence imaging of F30-2d×Broccoli-0×, 20×, 31× or 47× CAG repeats (0R, 20R, 31R, 47R)-tagged 100, 200, 500, 1000 or 2000 nt long scrambled RNA (0.1k, 0.2k, 0.5k, 1.0k and 2.0k), or OxyS (109 nt), *lacY* (1254 nt) and *lacZ* (3044 nt) RNA sequences. Fluorescence images were taken in solutions containing 4 μM RNA, 20 mM MgCl_2_ and 80 μM DFHBI-1T after annealing. Scale bar, 5 μm. (**B**) The mean droplet area and (**C**) partition ratio of individual condensates is plotted based on data shown in Supplementary Figures S5 and S6. (**D**) The radius and (**E**) partition ratio of each studied condensate is plotted as a function of the ratio between the number of CAG repeats and total RNA length. Results from each 20×, 31× or 47× CAG-tagged target RNA, including F30-2d×Broccoli, 0.1k, 0.2k, 0.5k, 1.0k and 2.0k scrambled RNAs, and OxyS, *lacY* and *lacZ* are gathered and denoted under 20R (cyan), 31R (light blue) and 47R (dark blue), respectively. Shown are the mean and the standard error of the mean (SEM) values from at least three replicated measurements for each data point.

Before testing RNA condensation in living cells, we also wondered if these CAG repeats can function as general molecular tags to induce the phase transition of attached RNA sequences. For this purpose, we synthesized 20 different RNA strands that contain 5′-F30-2d×Broccoli-tagged 100, 200, 500, 1000 or 2000 nucleotide (nt) long scrambled sequences. The 0×, 20×, 31× or 47× CAG repeats were conjugated at the 3′ end of these strands, respectively. The multiple of 4× CAG repeats was not chosen because condensation occurs with low efficiency. Without attaching CAG repeats, scrambled RNAs (named 0.1k, 0.2k, 0.5k, 1.0k and 2.0k) could not form condensates in a solution containing 4 μM RNA, 20 mM MgCl_2_ and 80 μM DFHBI-1T (Figure 2).

In contrast, the 20× CAG tag can induce compartmentalization when short RNA sequences (i.e. 100 and 200 nt) were attached, while obvious condensates were shown in all the solutions containing 31× CAG- or 47× CAG-tagged RNA strands. These results indicated that CAG repeats can still induce phase separation even after tagging with long non-condensation RNA strands.

We further quantified the correlations between the length of CAG repeats and the partition ratio and size of condensates. As shown in Supplementary Figure S5, a longer CAG-repeat tag can generally increase both the diameter and partition ratio of RNA condensates, while, interestingly, after attaching longer scrambled RNAs to the same CAG-repeat tag, the partition ratio and size of condensates tended to first increase and then decrease (Figure 2B, C). This result is consistent with those observed in synthetic multivalent polymers with changing valency of interactions (23), suggesting that the efficiency of condensate formation is influenced by the sequence length of both CAG repeats and target RNAs. A longer CAG-repeat tag is normally needed to recruit larger RNA targets into condensates.

Next, to explore if CAG repeats can also mediate condensation of endogenous RNA sequences, three bacterial RNAs were tested, namely a 109 nt long OxyS small non-coding RNA (sRNA) and two *lac* operon mRNAs, *lacY* (1254 nt) and *lacZ* (3044 nt). After tagging with F30-2d×Broccoli and 20×, 31× or 47× CAG repeats, OxyS can easily form obvious condensates, with size and partition ratio comparable with those of 0.1k scrambled RNAs (Figure 2; Supplementary Figure S6). Similarly, 31× and 47× CAG-tagged *lacY* mRNA can also generate condensates close to those of 1.0k scrambled RNAs. Even for the 3044 nt long *lacZ* mRNA, after conjugating with 31× CAG and 47× CAG, micrometer-sized condensates were clearly observed (Figure 2A). All these data indicated that the CAG-repeat tags can facilitate both scrambled and functional RNA species to partition into condensates.

By combining all these results from different RNA targets, our data showed that the size of condensates tended to be first enlarged and then reduced with an increasing ratio between the CAG repeat number and total RNA length, N_CAG_/N_total_ (Figure 2D). Meanwhile, the partition ratio is also generally increased at a low N_CAG_/N_total_ ratio and then slightly decreased at N_CAG_/N_total_ >5% for 31× and 47× CAG repeat samples (Figure 2E). These results suggested that the N_CAG_/N_total_ ratio can be potentially an important factor for regulating the aggregation status of RNA-repeat condensates.

### RNA condensation inside bacterial cells

**Figure 3.**
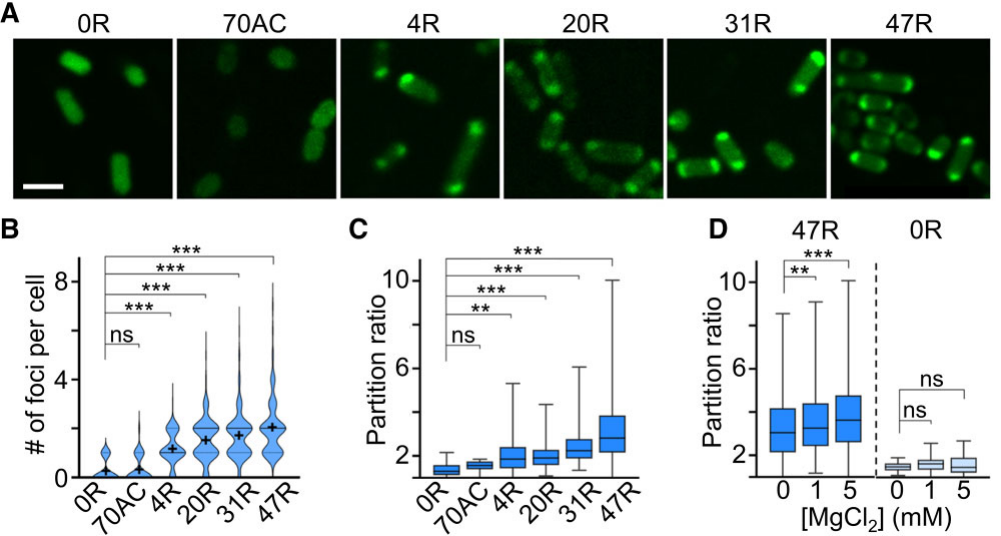
RNA condensate formation in living bacterial cells. (**A**) Confocal fluorescence imaging of BL21 Star™ (DE3) *E. coli* cells that express F30-2d×Broccoli-tagged 0×, 4×, 20×, 31×, 47× CAG repeats (0R, 4R, 20R, 31R, 47R) or 70× AC repeats (70AC). Scale bar, 2 μm. (**B**) The violin plot distribution of the number of foci per cell. Solid and dashed lines indicate the median and interquartile value, respectively. The black cross indicates the mean value. (**C**) The partition ratio of individual cellular foci as measured in DPBS buffer without adding MgCl_2_. A total of 20, 29, 241, 318, 323 and 437 condensates were analyzed in 0R, 70AC, 4R, 20R, 31R and 47R cells, respectively. (**D**) Magnesium ion-dependent partition ratio changes of individual cellular foci. A total of 421, 562 and 1058 (17,21,27) condensates in 47R (0R) cells were analyzed in the presence of 0, 1 or 5 mM Mg^2+^, respectively. Shown are box plots with minimum to maximum whiskers. The top and bottom line, upper and lower box boundary and inner line indicate the minimum and maximum data point excluding outliers, 75th and 25th percentile and median of the data, respectively. All the data are collected from at least three representative images. Two-tailed Student's *t*-test: ***P< 0.001; **P< 0.01; ns, not significant, *P*> 0.05.

After all these *in vitro* characterizations, we next asked if CAG repeats could also be used as genetically encoded tags to regulate the condensation of target RNAs inside living cells. In our test, we first transformed BL21 Star™ (DE3) *E. coli* cells with pET-28c vectors that express 4R, 20R, 31R or 47R sequences. Compared with the control cells encoding only F30-2d×Broccoli (0R) or 70AC, the formation of cellular condensates can be clearly visualized in CAG-repeat-containing strains (Figure 3A). Similar to the *in vitro* results, the number and partition ratio of cellular condensates highly depend on the length of CAG repeats. The most abundant and RNA-concentrated condensates were shown in 47R-expressing cells (Figure 3B, C).

The vast majority of CAG-repeat-expressing cells (4R–47R) contain one or two condensates (76–85%), mainly localized at the cell poles. Meanwhile, it is worth mentioning that minimal cytotoxicity was observed in these CAG repeat-expressing *E. coli* cells (Supplementary Figure S7). Indeed, CAG repeat-regulated formation of RNA condensates can occur in living bacterial cells.

We also applied FRAP to study the mobility of RNAs within these cellular condensates. In contrast to our *in vitro* data (Supplementary Figure S2), these intracellular RNA condensates exhibit more liquid-like properties as fast fluorescence recovery was observed: ∼90% of original fluorescence signals being reached in ∼30 s (Supplementary Figure S8). Interestingly, for cells possessing two major condensates at opposite poles, after photobleaching the condensate at one pole, a clear transfer of fluorescence signal from the other unbleached pole was observed in five out of nine tested cells (Supplementary Figure S8).

To further study if the formation of these cellular condensates can be simply regulated by adding magnesium ions to increase the binding affinities among CAG-repeat strands, we incubated 0R- and 47R-expressing cells with 0, 1 or 5 mM MgCl_2_. Indeed, both the number and partition ratio of RNA condensates were up-regulated in 47R cells after adding 5 mM MgCl_2_ (Figure 3D; Supplementary Figure S9). In contrast, no changes were observed in 0R cells; meanwhile, the average cellular Broccoli fluorescence in both groups of cells was not altered. Mg^2+^ can thus be used as a convenient regulator of these cellular CAG-repeat condensates.

In addition, our data showed that the formation of CAG condensates may reduce the cellular RNA degradation in these bacterial cells. In our test, after incubation for 24 h, ∼45% of 47R cellular fluorescence could still be observed, with most signals coming from condensates, while in 0R-expressing *E. coli* cells, the cellular fluorescence was decreased by >75% under this same experimental condition (Supplementary Figure S10).

We next tested if CAG repeats can also recruit other cellular RNA targets into condensates. For this purpose, we prepared pET-28c vectors that respectively express 0R-, 20R-, 31R- or 47R-tagged OxyS, *lacY* and *lacZ* RNAs. After transforming into BL21 Star™ (DE3) cells, bright fluorescent foci can be observed only in CAG-repeat-expressing cells (Supplementary Figure S11). Both the number and partition ratio of RNA condensates tend to be increased after attaching elongated CAG repeats (Supplementary Figure S11). On average, ∼1.5–2.4 condensates were shown in each individual cell, mostly at the poles. All these data supported that the CAG repeats can be used as genetically encoded tags to drive the phase transition of target RNAs inside living cells.

### Condensation-mediated cellular RNA regulation

**Figure 4.**
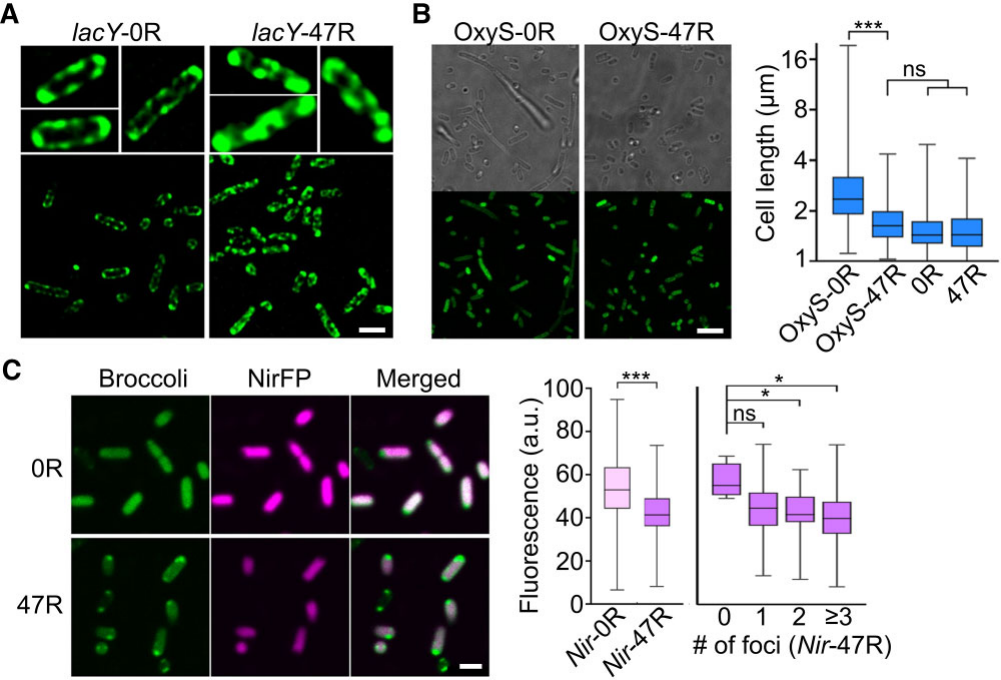
Condensation-regulated cellular localization and function of target RNAs. (**A**) Structured illumination microscope imaging of *E. coli* cells expressing 0R- or 47R-tagged *lacY* mRNA. Scale bar, 2 μm. (**B**) Confocal fluorescence imaging of *E. coli* cells expressing 0R- or 47R-tagged OxyS sRNA. Scale bar, 5 μm. Individual cell length is also plotted and compared with 0R- and 47R-expressing cells without attaching OxyS. Shown are box plots with minimum to maximum whiskers. A total of 200, 153, 221 and 201 cells were analyzed in OxyS-0R, OxyS-47R, 0R and 47R, respectively. (**C**) Confocal fluorescence imaging of *E. coli* cells expressing 0R- or 47R-tagged near-infrared fluorescent protein (NirFP) mRNA. Scale bar, 2 μm. Average cellular NirFP fluorescence as measured in 120 and 99 individual 0R- or 47R-tagged cells is plotted. Meanwhile, the NirFP fluorescence signals in individual 47R-tagged cells are also separately plotted based on the corresponding number of cellular foci. A total of 4, 25, 30 and 40 cells were analyzed with 0, 1, 2 and ≥3 foci, respectively. Shown are box plots with minimum to maximum whiskers. The top and bottom line, upper and lower box boundary and inner line indicate the minimum and maximum data point excluding outliers, 75th and 25th percentile and median of the data, respectively. All the data are collected from at least three representative images. Two-tailed Student's *t*-test: ***P< 0.001; *P< 0.05; ns, not significant, *P*> 0.05.

To further assess if the formation of CAG condensates will change the cellular localization of target RNAs, we applied super-resolution structured illumination microscopy to image 0R- and 47R-tagged *lacY* mRNA. It is known that *lacY* prefers to localize near bacterial membranes, i.e. the functioning site of its protein product, lactose permease LacY (24–26). Indeed, without the CAG-repeat modification, *lacY*-0R was exclusively located at the *E. coli* membranes, exhibiting a hollow fluorescent pattern around the cells (Figure 4A). In contrast, after tagging with 47R, the hollow membrane fluorescent pattern was disrupted and replaced by condensation across the cytoplasm in the majority of cells. These results further validated that the CAG-repeat tags can alter the cellular locations of the attached RNAs.

We also studied if the phase transition of target RNAs can be used to control their cellular functions. OxyS sRNA is known to repress the expression of transcription termination factor NusG and impair cell division, and as a result to generate long bacterial cells (27,28). By comparing the length of individual *E. coli* cells expressing either 0× CAG- or 47× CAG-tagged OxyS, our results showed that the average length of OxyS-47R cells is ∼46% shorter than that of OxyS-0R cells (Figure 4B). As a control, without attaching OxyS, 0R- and 47R-expressing *E. coli* cells exhibited almost identical lengths to those of OxyS-47R cells. These data indicated that after condensation, the regulatory function of OxyS on cell division is impaired, probably due to a reduced chance of OxyS to interact with *nusG* mRNA, which resumed the NusG expression (27).

To further test if protein synthesis can indeed be regulated via the condensation of specific mRNAs, we transformed BL21 Star™ (DE3) cells with vectors expressing 0× CAG- or 47× CAG-tagged 705 nt long mRNA (named *Nir*-0R and *Nir*-47R) that encode a near-infrared fluorescent protein (NirFP). Compared with *Nir*-0R cells, ∼20% lower NirFP signals were observed in *Nir*-47R cells (Figure 4C). Most *Nir*-47R RNA was located within condensates at the poles (as shown in the Broccoli channel), while the majority of translated NirFP proteins were observed throughout the cells, except the poles. We further plotted the cellular NirFP signals as a function of the number of condensates in each *Nir*-47R cell. A reduced NirFP signal was shown in cells that contain more condensates (Figure 4C). These data suggested that gene expression can possibly be regulated by the CAG-repeat-mediated condensation of cellular mRNAs.

### *Trans*-acting RNA condensation tags

**Figure 5.**
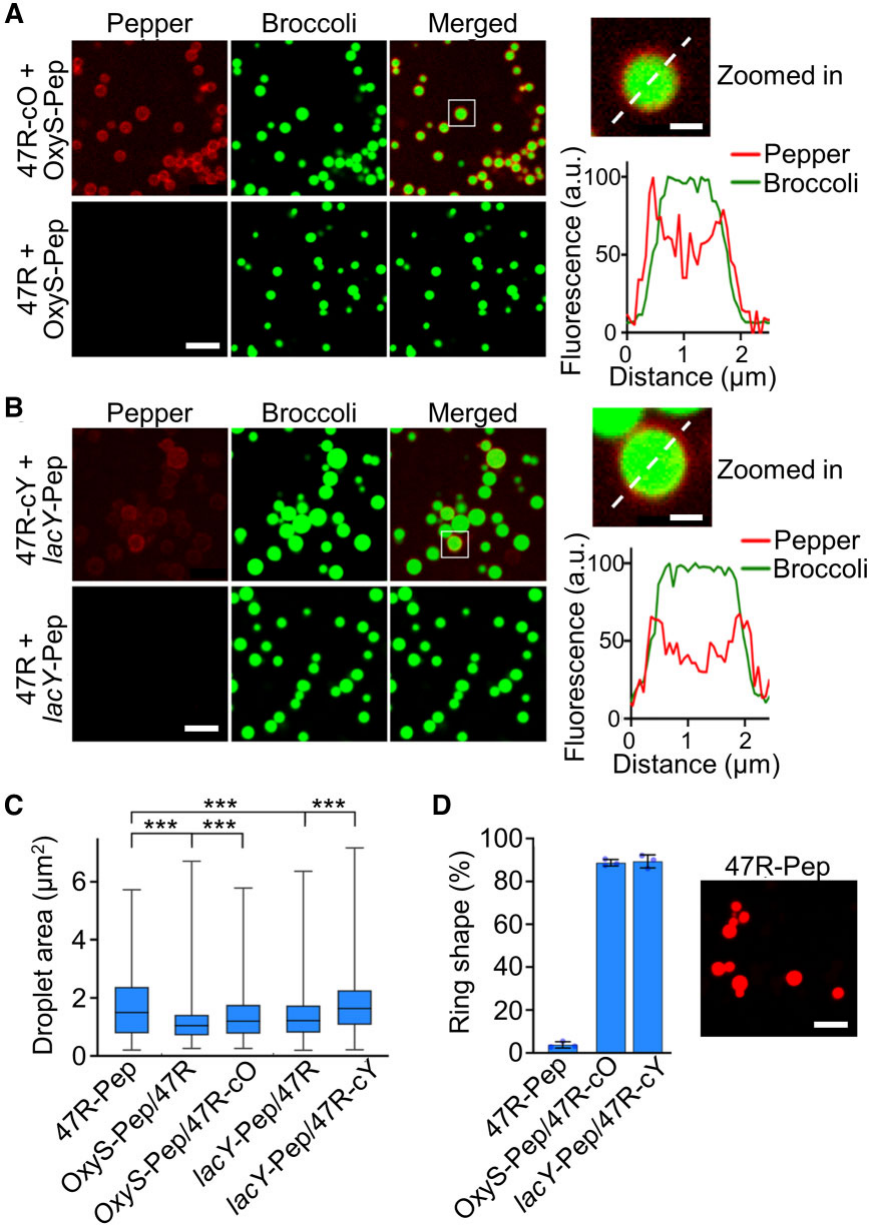
Targeted RNA recruitment into condensates via *trans*-acting CAG repeats. (**A** and **B**) Confocal fluorescence imaging of condensates induced by F30-2d×Broccoli-tagged 47× CAG repeats, with or without a complementary strand that targets OxyS or *lacY* (47R-cO, 47R-cY, 47R). Pepper-tagged OxyS and *lacY* were used as target RNAs (OxyS-Pep, *lacY*-Pep). Insets show an enlarged view of condensate. Broccoli (green) and Pepper (red) fluorescence were plotted based on distance across the dashed line. Fluorescence images were taken in solutions containing 4 μM of each RNA, 20 mM MgCl_2_, 2 μM HBC620 and 80 μM DFHBI-1T after annealing. Scale bar, 5 μm. Inset scale bar, 1 μm. (**C**) Droplet area of each type of RNA condensate is plotted in box and minimum to maximum whiskers. The top and bottom line, upper and lower box boundary and inner line indicate the minimum and maximum data point excluding outliers, 75th and 25th percentile and median of the data, respectively. (**D**) The percentage of ring-shaped condensates was measured in OxyS-Pep/47R-cO and *lacY*-Pep/47R-cY solutions, as compared with that of 47R-Pep. A total of 780–1010 condensates were analyzed in each case. Fluorescence imaging of condensation of Pepper-tagged 47× CAG repeats formed in solutions containing 4 μM RNA, 20 mM MgCl_2_ and 2 μM HBC620. Scale bar, 5 μm. Shown are the mean and SD values. Data were collected from at least three representative images. Two-tailed Student's *t*-test: ***P< 0.001.

We next wanted to study if these CAG repeats can be used as *trans*-acting effectors to potentially control the cellular condensation of endogenous target RNAs. To test this, we first *in vitro* synthesized F30-2d×Broccoli-containing 47R strands (named 47R-cO and 47R-cY) that were tagged with a complementary sequence that hybridizes with either an OxyS or *lacY* target RNA. A 23 nt and a 26 nt long non-structural region in OxyS and *lacY* was respectively designed as the targeting domain, whose secondary structures were pre-evaluated via Mfold and NUPACK software (29,30). To image the proposed RNA–RNA interactions, a secondary fluorogenic RNA aptamer (Pepper) (31) was tagged to the target OxyS and *lacY* RNAs, i.e. OxyS-Pep and *lacY*-Pep. After mixing OxyS-Pep with 47R-cO (Figure 5A) or *lacY*-Pep with 47R-cY (Figure 5B), the fluorescence signals from target RNAs (Pepper channel) were clearly accumulated and co-localized with the 47R condensates (Broccoli channel). As a negative control, without attaching the complementary sequence, 47R strands can still form obvious condensates but without recruiting OxyS-Pep or *lacY*-Pep (Figure 5A, B). Förster resonance energy transfer (FRET) between the Broccoli/DFHBI-1T (donor) and Pepper/HBC620 (acceptor) pair was also observed in the condensates formed by mixing 47R-cO with OxyS-Pep (Supplementary Figure S12). These FRET signals further support the spatial proximity of these complementary strands and the occurrence of RNA–RNA interactions.

Interestingly, a ring-shaped Pepper fluorescence pattern was observed in OxyS-Pep/47R-cO and *lacY*-Pep/47R-cY samples (Figure 5A, B). Meanwhile, the recruitment of target RNAs led to an increase in the condensate size (Figure 5C). These results suggested that the target RNAs were mainly hybridized to the surface areas of condensates, probably after the initial formation of the condensate core regions. To further assess if this ring-shaped fluorescence distribution could have resulted from the misfolding of Pepper RNAs inside the center of condensates, we synthesized a control strand with Pepper directly tagged with a 47× CAG repeat (47R-Pep). 47R-Pep exhibited minimal ring-shaped condensates (Figure 5D), indicating that the ring-shaped Pepper fluorescence in OxyS-Pep/47R-cO and *lacY*-Pep/47R-cY samples was indeed likely to be due to the surface attachment of these target RNAs onto the CAG-repeat condensates.

**Figure 6.**
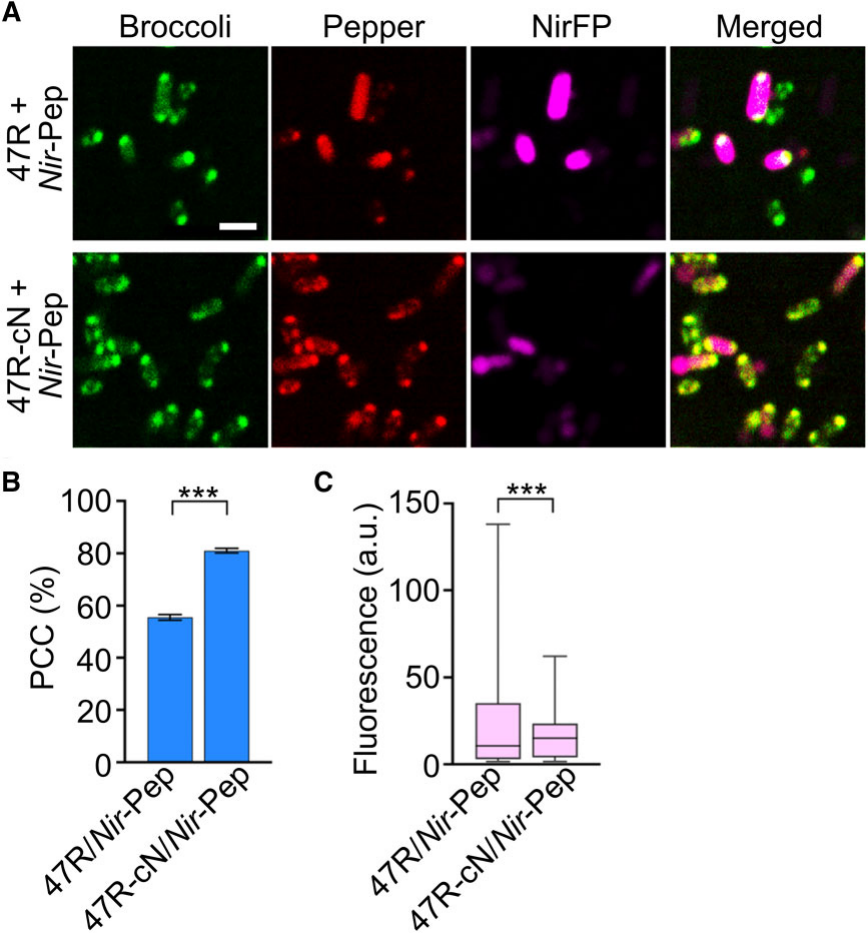
*Trans*-acting recruitment and functional regulation of target RNAs. (**A**) Confocal fluorescence imaging of *E. coli* cells that express Pepper-tagged near-infrared fluorescent protein RNA (*Nir*-Pep) and F30-2d×Broccoli-tagged 47× CAG repeats with or without a complementary targeting pair (47R-cN, 47R). Scale bar, 2 μm. (**B**) The Pearson's correlation coefficient (PCC) of the Broccoli and Pepper fluorescence channels was quantified for both 47R and 47R-cN cells in the presence of 1 μM HBC620 and 5 mM Mg^2+^. A total of 1287 and 1764 cells were analyzed in each case. Shown are the mean and SEM values. (**C**) Average cellular NirFP fluorescence as measured in individual 47R or 47R-cN cells is plotted in box and minimum to maximum whiskers. The top and bottom line, upper and lower box boundary and inner line indicate the minimum and maximum data point excluding outliers, 75th and 25th percentile and median of the data, respectively. A total of 717 and 742 cells were analyzed in each case. Data were collected from at least three representative images. Two-tailed Student's *t*-test: ***P< 0.001.

Lastly, we tested if these *trans*-acting CAG-repeat tags can also function inside living cells. For this purpose, we first imaged 47R-Pep fluorescence signals inside BL21 Star™ (DE3) cells. Similar to that shown in Broccoli-tagged CAG-repeat-expressing cells (Figure 3A), ∼62% of 47R-Pep-expressing cells also contain one or two condensates at the poles (Supplementary Figure S13A). Next, we transformed BL21 Star™ (DE3) cells with vectors expressing Pepper-tagged *NirFP* mRNA (*Nir*-Pep) together with an F30-2d×Broccoli-conjugated 47× CAG-repeat strand (47R) or that contains a 25 nt long complementary sequence 47R-cN. Despite some spectral overlaps, the NirFP fluorescence and Pepper/HBC620 signals can still be imaged simultaneously with negligible cross-talk (Supplementary Figure S13). In the presence of 5 mM MgCl_2_, based on the Pearson's correlation coefficient of the two fluorescent channels, ∼80% of Pepper signals in *Nir*-Pep- and 47R-cN-expressing cells were co-localized with CAG-repeat condensates (Figure 6A, B), while, in contrast, only 55% of *Nir*-Pep RNAs were found inside 47R condensates, indicating the roles of complementary sequence in recruiting target RNAs into CAG-repeat condensates. Consistent with the results from *cis-*acting CAG-repeat tags (Figure 4C), after forming condensates, ∼30% lower NirFP signals were observed in 47R-cN/*Nir*-Pep cells as compared with that in the 47R/*Nir*-Pep control (Figure 6C). The same 47R-cN/*Nir-*Pep vector was also used to express these *trans*-acting CAG-repeat tags in *P. aeruginosa* cells that carry an inducible, chromosomally integrated T7 RNA polymerase (32). Indeed, compared with 0R-cN/*Nir-*Pep-expressing *P. aeruginosa*, significantly more 47R-cN/*Nir-*Pep cells contain condensates, also with a much higher condensate partition ratio (Supplementary Figure S14). All these data suggested that *trans*-acting CAG-repeat tags can be used to recruit cellular mRNAs into condensates, which may be potentially engineered into a functional gene regulation platform.

## DISCUSSION

The importance of RNA condensation in studying cellular functions as well as disease diagnosis and treatment has been increasingly recognized (11,33). Programmable and self-functional probes that enable precise cellular RNA condensate regulation are thus useful tools in the field of chemical biology and synthetic biology. In this study, we demonstrated that naturally existing CAG repeats can be used as genetically encoded tags to induce cellular condensation of different RNAs of interest, including small non-coding RNAs and long mRNAs (>3000 nt). The formation of these self-assembled RNA condensates can be tracked *in vitro* and in living cells by fluorogenic RNA aptamers. The number, size and density of RNA condensates can be easily regulated by the length of CAG-repeat tags and Mg^2+^ concentration. The cellular RNA localization and compartmentalization can be rationally tuned by these CAG-repeat tags, via either a *cis-* or a *trans*-acting mechanism. Critically, the cellular functions of these target RNAs, such as in RNA–protein interactions (in the case of *lacY*) and gene expression patterns (in the case of *NirFP* and OxyS), can also be regulated by these CAG-repeat tags.

We expect that these functional CAG-repeat tags can be broadly applied to reprogram living cells with defined compartmentalization and structures. This work can also inspire the potential engineering of various types of genetically encodable RNA nanodevices, which can be based on similar nature-inspired RNA repeat tags. These tags will be used to regulate different endogenous RNA targets and may also be applied to orthogonally recruit different RNA and protein molecules into specific RNA condensates. Powerful nanodevices may also possibly be designed to be controllable by different small-molecule or light triggers.

## Supplementary Material

gkad621_Supplemental_File

## Data Availability

The data underlying this article will be shared on reasonable request to the corresponding author.
