# Supplementary material for: Targeted RNA condensation in living cells via genetically encodable triplet repeat tags
Source: Nucleic Acids Res. 2023 Jul 24;51(16):8337–47. doi: 10.1093/nar/gkad621 (PMC10484661; doi:10.1093/nar/gkad621)
Supplement: gkad621_Supplemental_File [file gkad621_supplemental_file.pdf]

Supplementary Information

Targeted RNA Condensation in Living Cells via Genetically Encodable Triplet Repeat Tags

Zhaolin Xue,[1,+] Kewei Ren,[1,3,+,*] Rigumula Wu,[1] Zhining Sun,[1] Ru Zheng,[1] Qian Tian,[1] Ahsan Ausaf Ali,[1] Lan Mi[1] and Mingxu You[1,2,*]

[1] Department of Chemistry, University of Massachusetts, Amherst, MA 01003, USA
[2] Molecular and Cellular Biology Program, University of Massachusetts, Amherst, MA 01003, USA
[3] School of Chemistry and Chemical Engineering, Nanjing University of Science and Technology, Nanjing, 210094, China

* Correspondence: mingxuyou@chem.umass.edu and kwren@njust.edu.cn
[+] These authors contribute equally.

The Supplementary Information includes:

Reagents and apparatus
Supplementary Figure S1 to S14
Supplementary Table S1

Reagents and apparatus

DNA oligonucleotides used in this work were synthesized and purified by Integrated DNA Technologies (Coralville, IA) and W. M. Keck Oligonucleotide Synthesis Facility (Yale University School of Medicine). The detailed sequences have been listed in Table S1. PCR products were cleaned using a Monarch® PCR & DNA Cleanup Kit [New England Biolabs (NEB), Ipswich, MA]. All the RNAs for in vitro experiments were transcribed using a HiScribe™ T7 high-yield RNA synthesis kit (New England Biolabs, Ipswich, MA) and purified with G-25 columns. All chemicals were of analytical grade and obtained from Sigma or Fisher Scientific unless otherwise noted. All the concentrations of nucleic acids were measured with a NanoDrop One UV-Vis spectrophotometer. The gel electrophoresis was performed on a BioRad electrophoresis analyzer (Bio-Rad, Hercules, CA) and imaged on a Bio-Rad Gel Doc EZ imager. Fluorescence measurements in solution were conducted with a BioTek Synergy 2 fluorescence plate reader (λ_{ex}= 485/20 nm, λ_{em}= 528/20 nm) (BioTek, Winooski, VT).

Supplementary Figures

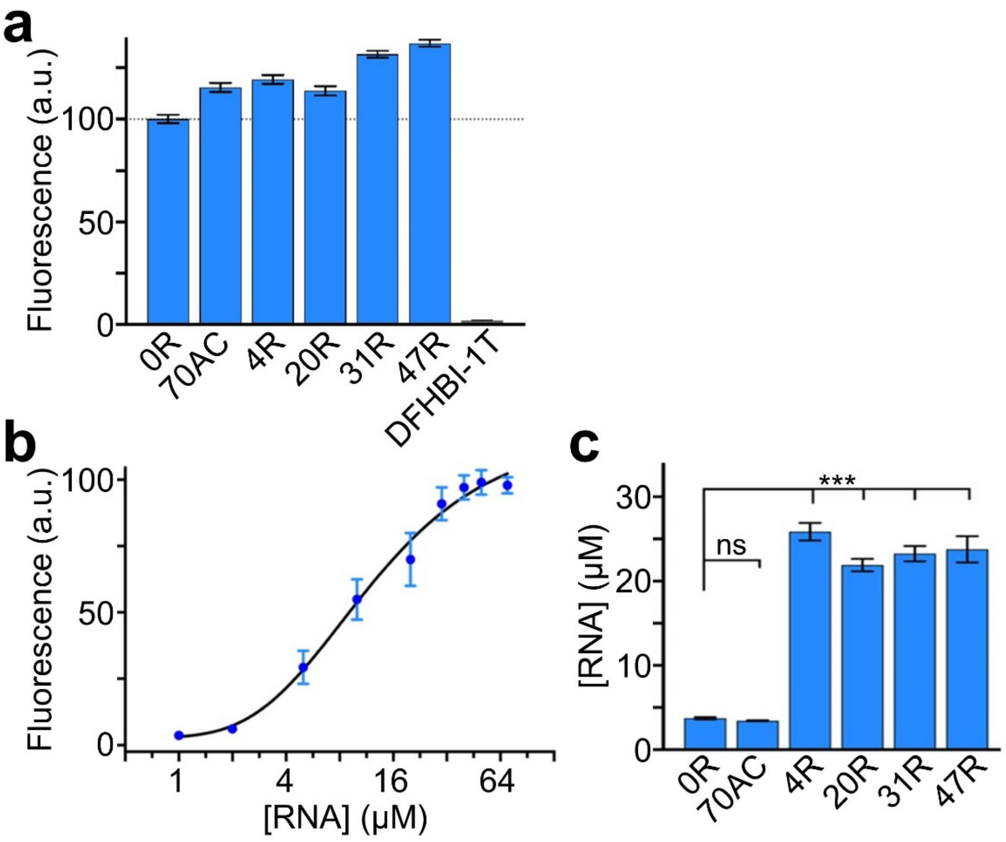

Figure S1. (**a**) Fluorescence measurement as performed in solutions containing 4 μM corresponding RNA, 20 mM $MgCl_2$, and 80 μM DFHBI-1T after annealing for the *in vitro* RNA condensate formation. F30-2d×Broccoli-tagged RNA strands containing 0×, 4×, 20×, 31×, 47×CAG repeats or 70×AC repeats are depicted as 0R, 4R, 20R, 31R, 47R, and 70AC, respectively. Shown are the mean and standard deviation (SD) values from three replicated experiments. (**b**) The correlation between RNA concentration and fluorescence signal intensities as measured in solutions containing 0–70 μM F30-2d×Broccoli RNA, 20 mM $MgCl_2$, and 80 μM DFHBI-1T under the same imaging condition for the *in vitro* RNA condensation characterization. Shown are the mean and SD values from three replicated experiments. (**c**) Based on the calibration curve shown in the panel (b), the estimated RNA concentration within each condensate formed in Figure 1. Shown are the mean and the standard error of the mean (SEM) values from three representative images. Two-tailed student's t-test: ***, $p<0.001$; ns, not significant, $p>0.05$.

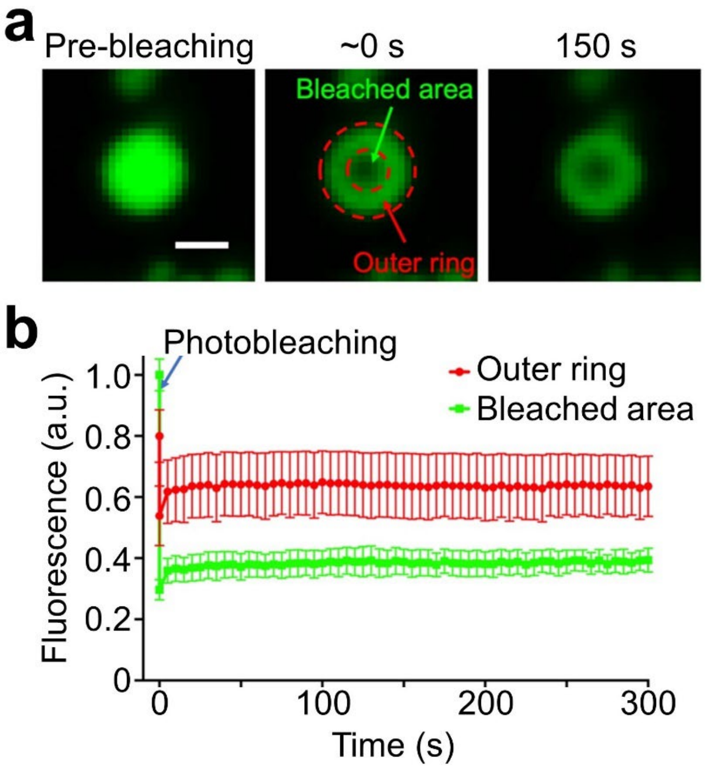

Figure S2. (**a**) *In vitro* fluorescence recovery after photobleaching (FRAP) measurement within condensates formed in solutions containing 4 μM 47R RNA, 20 mM $MgCl_2$, and 80 μM DFHBI-1T. Green and red arrows indicate the bleached area and outer ring region, respectively. Scale bar, 1 μm. (**b**) Averaged fluorescence intensity as measured within the bleached area (green line) and the outer ring region (red line) and plotted over time for the FRAP measurement. Shown are the mean and SEM values from at least three representative condensate FRAP measurements.

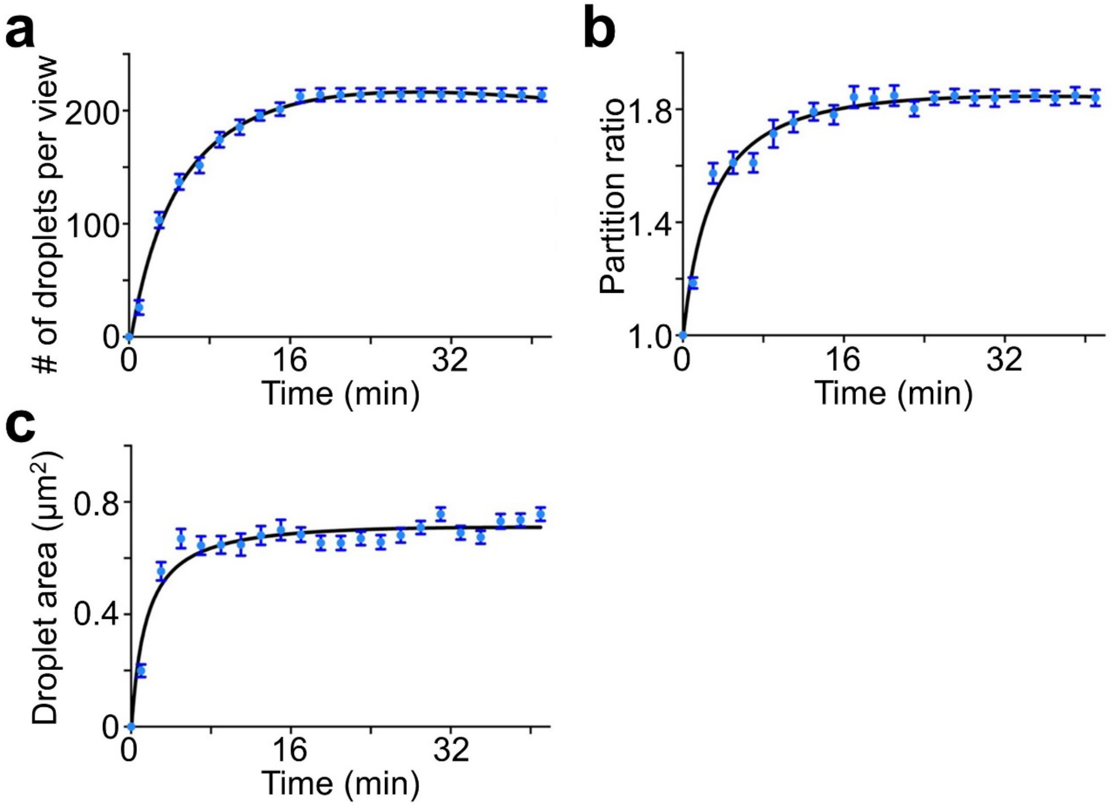

Figure S3. *In vitro* kinetic measurement of the condensate formation. (**a**) The number of condensates per imaging view, (**b**) the partition ratio and (**c**) area of each condensate are plotted after a 3 min 95°C heating and 30 s ice cooling. The condensate solution contains 4 μM 47R RNA, 20 mM $MgCl_2$, and 80 μM DFHBI-1T. The partition ratio is defined as the ratio of average fluorescence intensity inside individual condensate versus background signals in the solution. Shown are the mean and SEM values from at least three representative images, each imaging view equals to 4,430 μm^2.

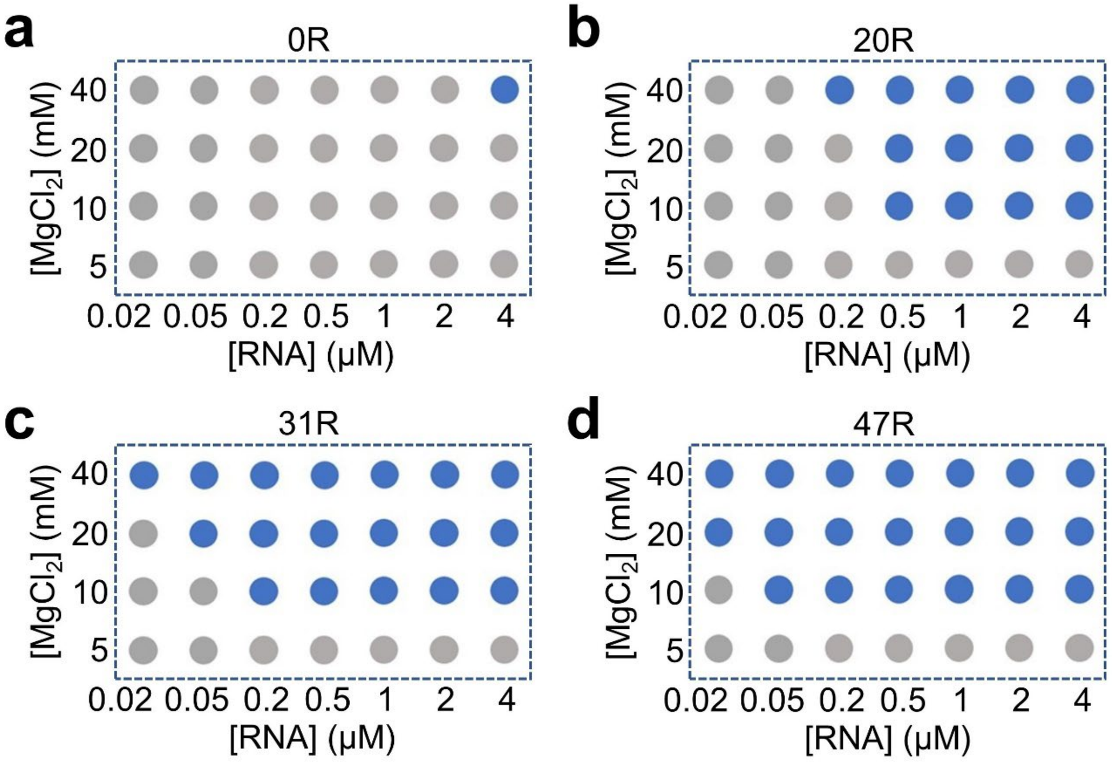

Figure S4. The phase diagrams of Mg^{2+} and RNA concentration-dependent *in vitro* condensate formation. The measurements were performed in a solution after annealing for the condensate formation, which contains 10 mM Tris-HCl at pH=7.5, 100 mM KCl, and DFHBI-1T of 20-fold concentration as that of RNA. Blue (gray) dots represent the presence (absence) of >10 condensate with partition ratio >2 per 4,430 μm^2 imaging view from at least three replicated experiments.

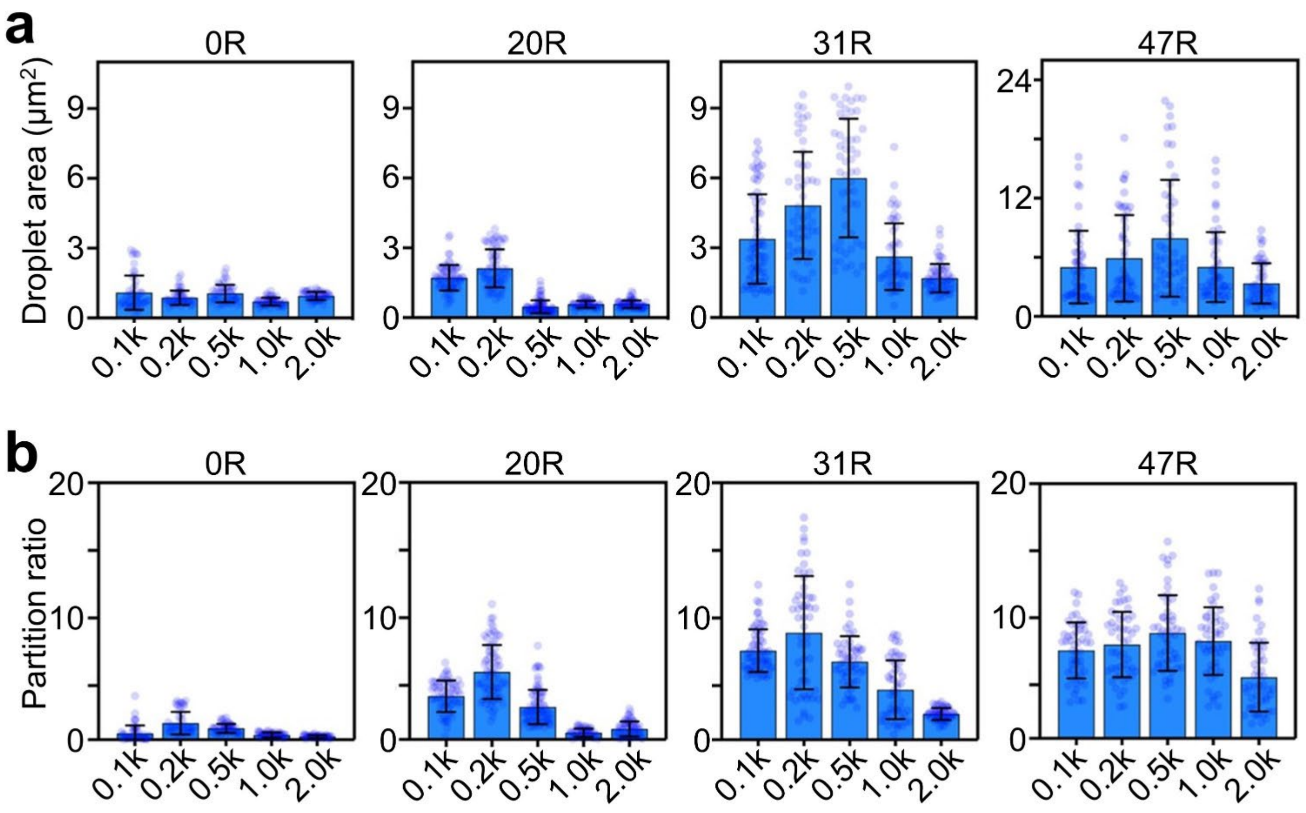

Figure S5. *In vitro* characterization of CAG-repeat-mediated condensation of different scrambled RNAs. (**a**) The area and (**b**) partition ratio of each condensate is plotted as a function of the length of scrambled target RNAs. The measurement was performed in solutions containing 4 μM corresponding RNA, 20 mM $MgCl_2$, and 80 μM DFH BI-1T after annealing for the condensate formation. Each data point represents one condensate. Shown are the mean and SD values from at least three representative images, each imaging view equals to 4,430 $μm^2$.

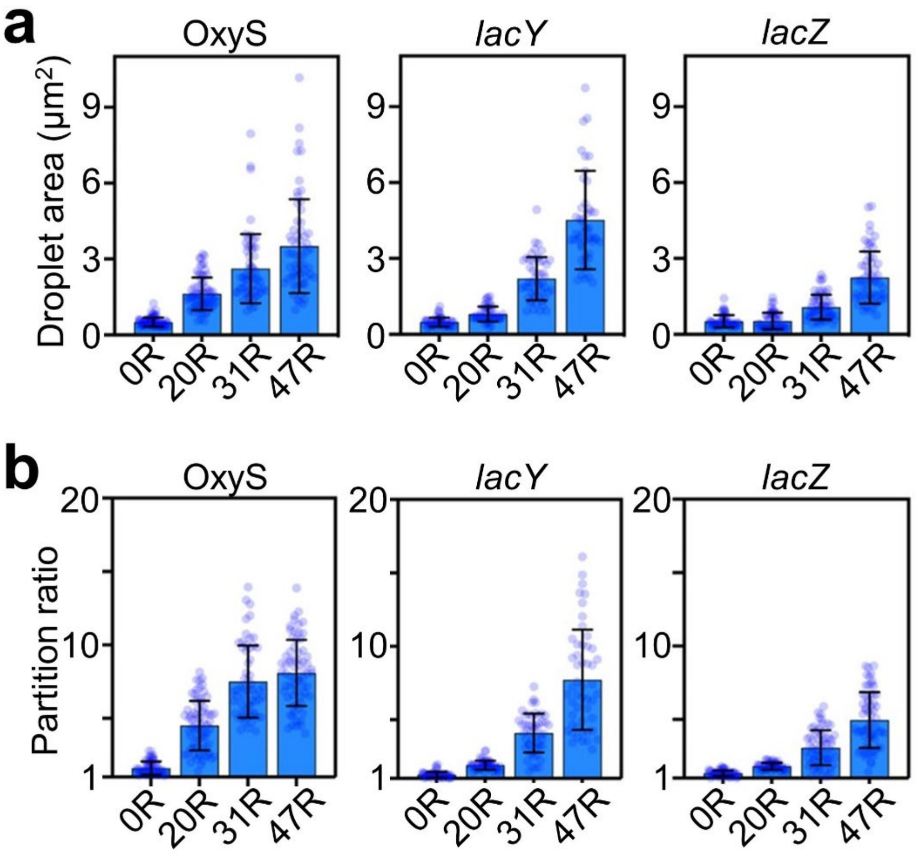

Figure S6. *In vitro* characterization of CAG-repeat-mediated condensation of different target RNAs. (**a**) The area and (**b**) partition ratio of each condensate is plotted as a function of the length of CAG repeats for each target RNAs. The measurement was performed in solutions containing 4 μM corresponding RNA, 20 mM $MgCl_2$, and 80 μM DFHBI-1T after annealing for the condensate formation. Each data point represents one condensate. Shown are the mean and SD values from at least three representative images, each imaging view equals to 4,430 μm^2.

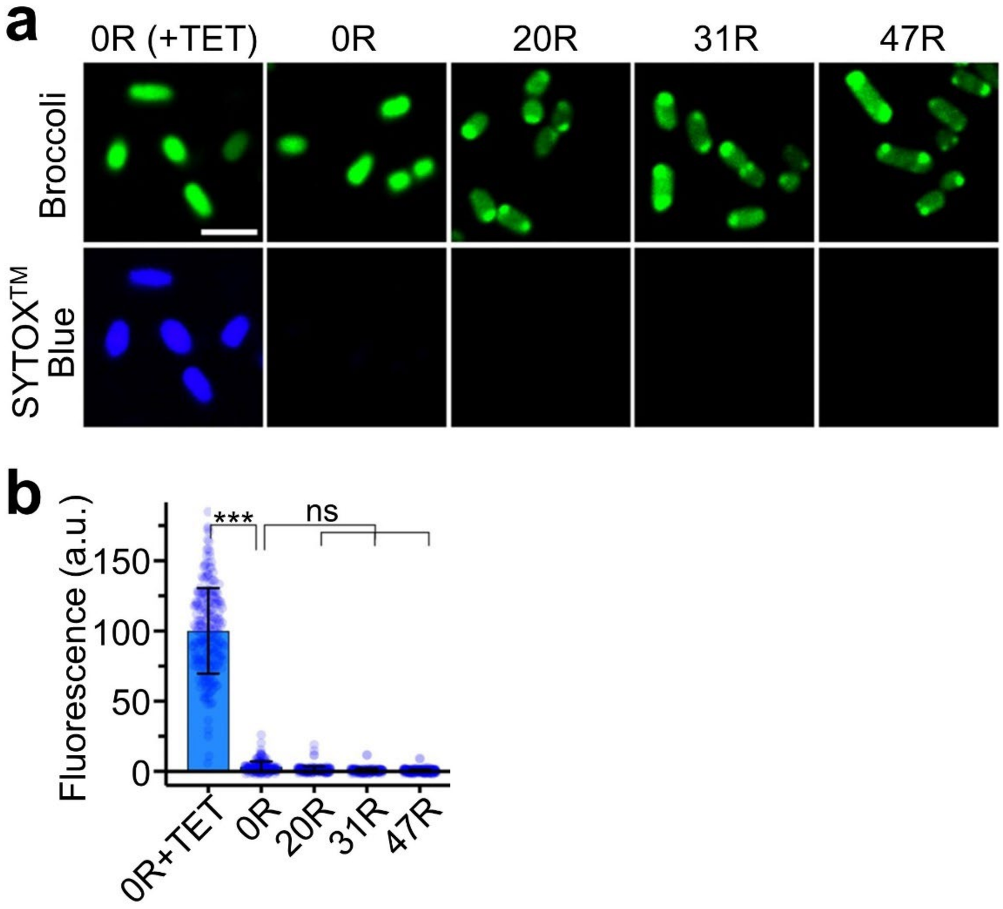

Figure S7. The cytotoxicity assessment of CAG-repeat-expressing bacterial cells. (**a**) Fluorescence imaging of BL21 Star™ (DE3) *E. coli* cells that express a pET-28c-F30-2d×Broccoli-0×, 20×, 31× or 47×CAG (0R, 20R, 31R or 47R) plasmid. These cells were incubated in DPBS buffer containing 1 μM SYTOX™ Blue for 2 h before imaging. As a positive control, one well of 0R cells were also treated with 1 mM tetracycline (TET) during the 2 h incubation with the SYTOX™ Blue dye. Scale bar, 3 μm. (**b**) Average cellular SYTOX™ Blue fluorescence as measured in individual cells. Each data point represents one cell. Shown are the mean and SD values. All the data is collected from at least three representative images. Two-tailed student's t-test: ***, $p<0.001$; ns, not significant, $p>0.05$.

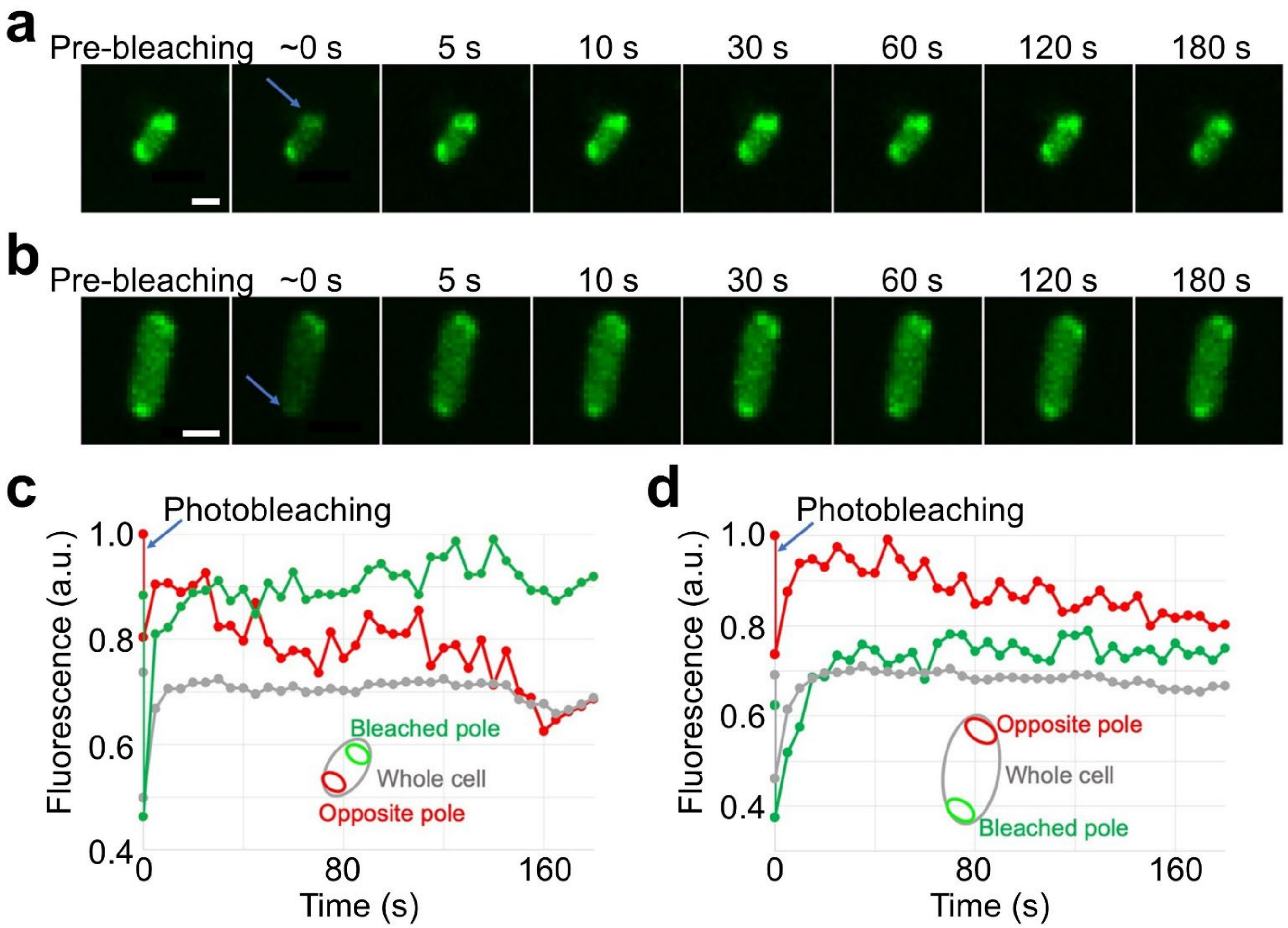

Figure S8. The FRAP measurement in BL21 Star^{TM} (DE3) *E. coli* cells that express a pET-28c-F30-2d×Broccoli-47×CAG plasmid (47R). (**a**, **b**) Representative FRAP images from five out of nine measured cells containing two condensates at opposite poles. All these five cells show the transfer of fluorescence signals between two poles after photobleaching. Blue arrows indicate the bleached pole. Scale bar, 1 μm. (**c**, **d**) Averaged fluorescence intensity from the bleached pole (green dot/line), unbleached opposite pole (red dot/line) and whole cell (grey dot/line) are plotted over time for the FRAP measurement. The insets illustrate the corresponding measured regions.

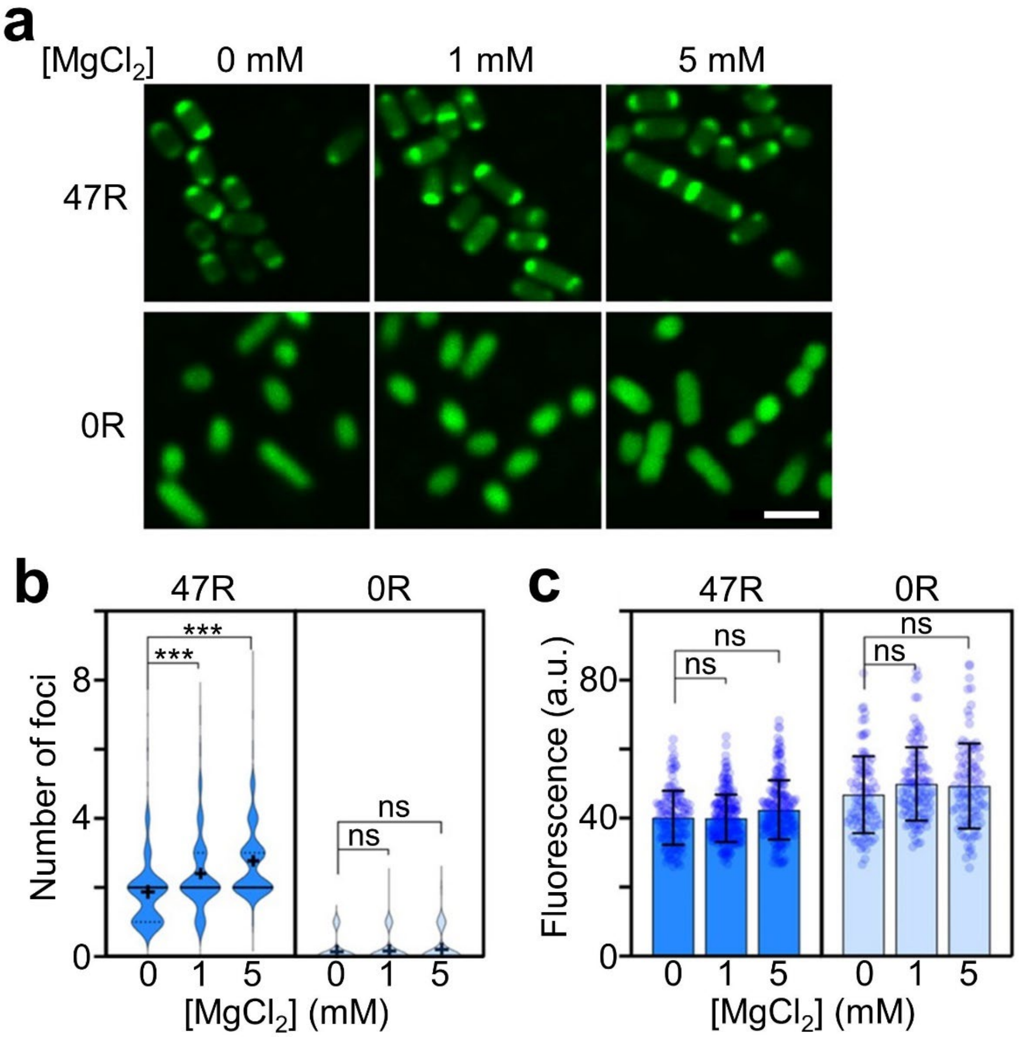

Figure S9. Mg^{2+} concentration-regulated cellular RNA condensate formation. (**a**) Fluorescence imaging of BL21 Star^{TM} (DE3) *E. coli* cells that express a pET-28c-F30-2d×Broccoli-0×CAG (0R) or pET-28c-F30-2d×Broccoli-47×CAG (47R) plasmid. These cells were incubated in DPBS buffer containing 0, 1, or 5 mM $MgCl_2$ for 2 h before imaging. Scale bar, 2 μm. (**b**) The violin plot distribution of the number of foci per cell, as measured from 0R or 47R cells. Solid and dashed line indicate the median and interquartile value, respectively. The black cross indicates the mean value. (**c**) The averaged fluorescence intensity as measured in each individual cell. Each data point represents one cell. Shown are the mean and SD values. All the data is collected from at least three representative images. Two-tailed student's t-test: ***, $p<0.001$; ns, not significant, $p>0.05$.

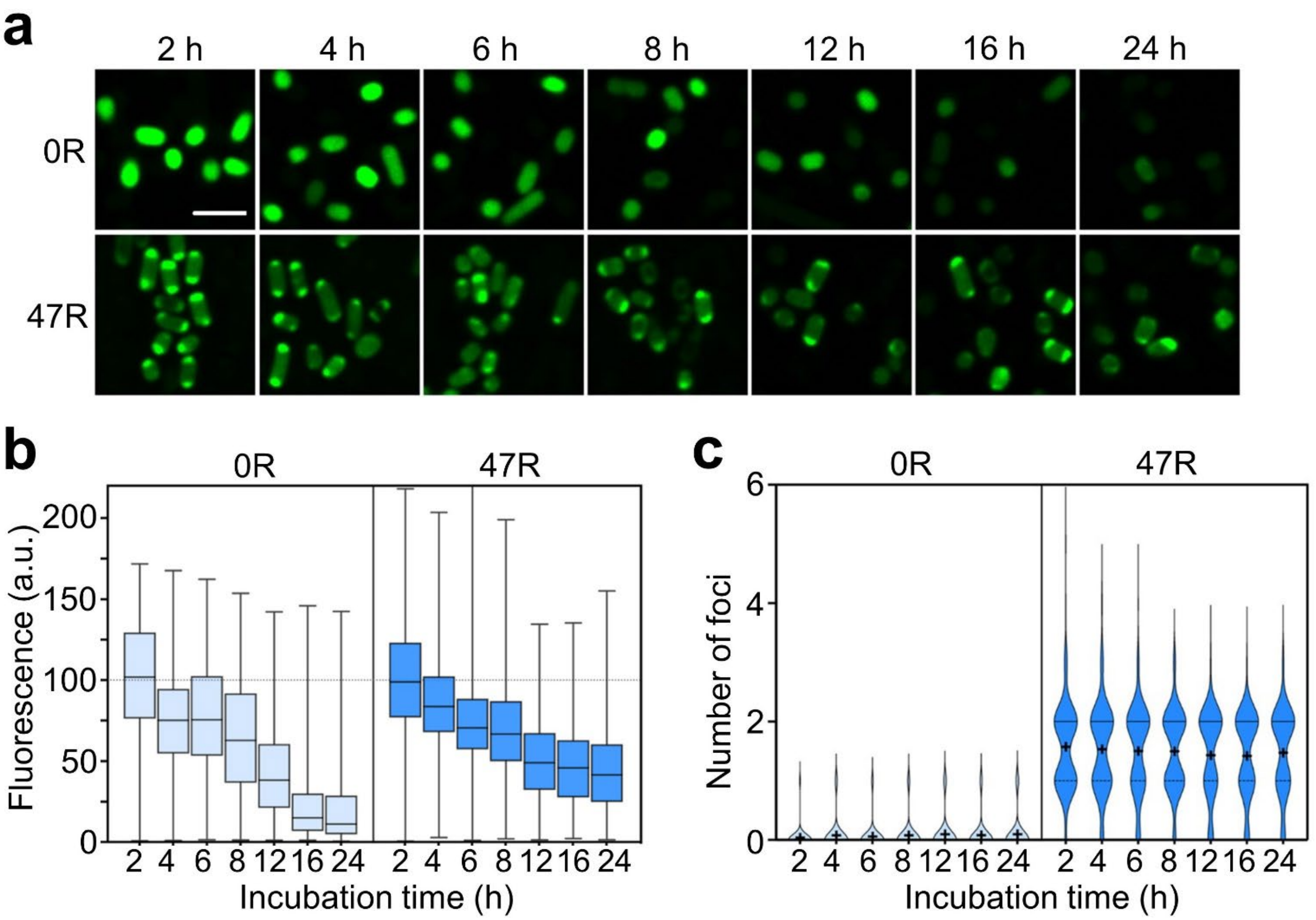

Figure S10. Condensate formation-mediated changes in cellular RNA lifetime. (**a**) Fluorescence imaging over 24 hours of BL21 Star™ (DE3) *E. coli* cells that express a pET-28c-F30-2d×Broccoli-0×CAG (0R) or pET-28c-F30-2d×Broccoli-47×CAG (47R) plasmid. These cells were first IPTG-induced for 2 h and then after removing the IPTG, left in the DPBS buffer for different time before imaging. Scale bar, 3 μm. (**b**) The averaged fluorescence intensity as measured in each individual cell. Shown are box plots with min-to-max whiskers collected from at least three representative images. The top and bottom line, upper and lower box boundary, and inner line indicate the minimum and maximum data point excluding outliers, 75th, 25th percentile, and median of the data, respectively. (**c**) The violin plot distribution of the number of foci per cell, as measured from 0R or 47R cells after different time of incubation in the DPBS buffer. Solid and dashed line indicate the median and interquartile value, respectively. The black cross indicates the mean value. All the data is collected from at least three representative images.

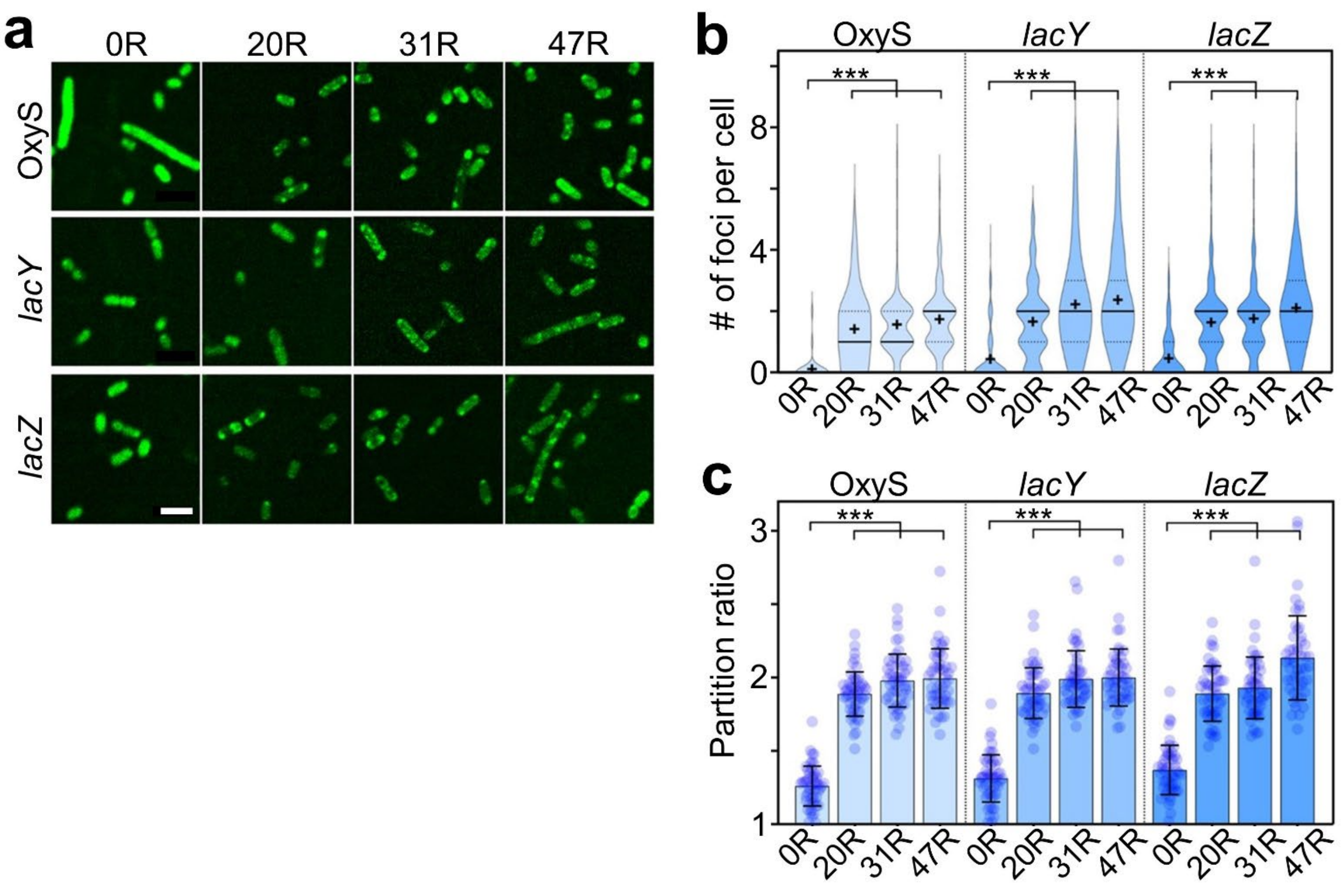

Figure S11. Condensation of target RNAs in *E. coli* cells. (**a**) Fluorescence imaging of BL21 Star^{TM} (DE3) *E. coli* cells that express a pET-28c vector encoding 0R-, 20R-, 31R-, or 47R-tagged OxyS, *lacY* or *lacZ* target RNAs. Scale bar, 5 μm. (**b**) The violin plot distribution of the number of foci per cell, as measured from corresponding cells. Solid and dashed line indicate the median and interquartile value, respectively. The black cross indicates the mean value. (**c**) The partition ratio of individual cellular foci as measured from the same batch of cells. Each data point represents one cellular condensate. Shown are the mean and SD values. All the data is collected from at least three representative images. Two-tailed student's t-test with Bonferroni correction: ***, $p < 0.0003$.

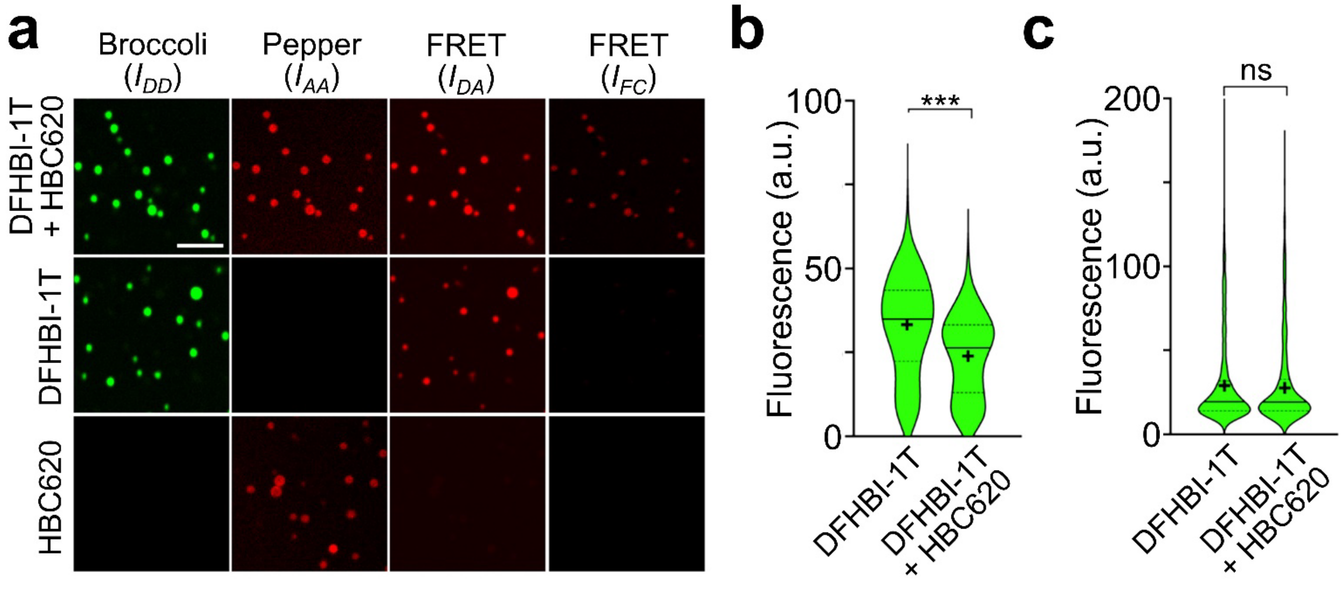

Figure S12. Förster resonance energy transfer (FRET) in trans-acting RNA condensates. (**a**) Confocal fluorescence imaging of condensates induced by a mixture of 47R-cO and OxyS-Pep. Images were acquired using three different channels, i.e., Broccoli channel (I_{DD}, excitation: 488 nm, emission: 505–535 nm), Pepper channel (I_{AA}, excitation: 561 nm, emission: 580–620 nm), and FRET channel (I_{DA}, excitation: 488 nm, emission: 580–620 nm). Samples were imaged respectively in solution containing only 80 μM DFHBI-1T (donor), only 2 μM HBC620 (accepter) or both (FRET). The corrected FRET (I_{FC}) was calculated following previously reported procedures[1-3]: $I_{FC} = I_{DA} - aI_{AA} - dI_{DD}$, where the cross-talk parameters d, a were determined based on donor only or accepter only conditio, i.e., in this case, $d = I_{DA}^{donor}/ I_{DD}^{donor}$ = 33.4% and $a = I_{DA}^{accepter}/ I_{AA}^{accepter}$ =12.1%. Scale bar, 5 μm. (**b**) Average Broccoli fluorescence signals as measure in 581 and 392 individual condensates were plotted from (a) with samples containing 80 μM DFHBI-1T in the absence or presence of 2 μM HBC620, respectively. The FRET efficiency (E) can be estimated based on the quenching of donor signal as $E = (I_{DD}^{donor} - I_{DD}^{FRET}) / I_{DD}^{donor}$ = 29%. (**c**) Average cellular foci Broccoli fluorescence signals as measured in 47R-cN/*Nir*-Pep expressing cells from Figure 6. A total of 795 and 577 individual cells were plotted with DFHBI-1T before and after adding HBC620, respectively. Shown are violin plots with solid and dashed line indicating the median and interquartile value, respectively. The black cross indicates the mean value. Data was measured from at least three representative images. Two-tailed student's t-test: ***, $p<0.001$; ns, not significant, $p>0.05$.

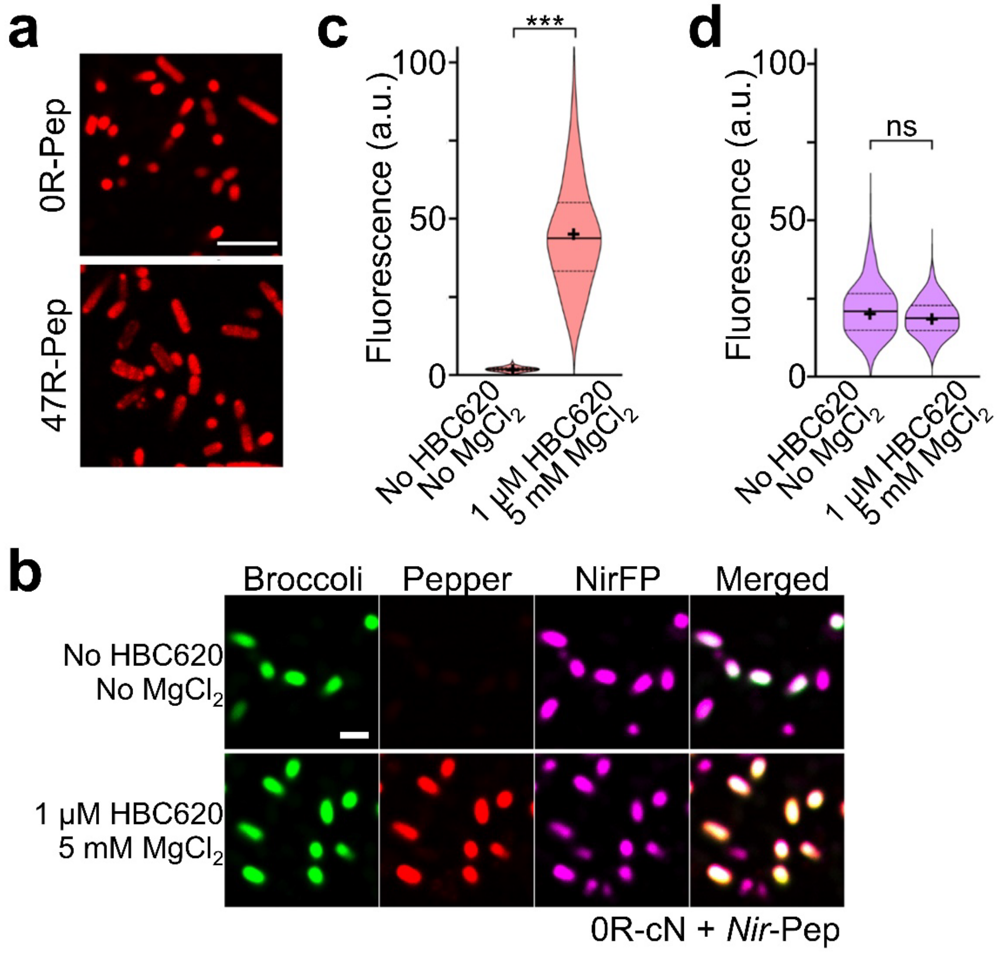

Figure S13. Trans-acting recruitment and regulation of target cellular RNAs. (**a**) Fluorescence imaging of BL21 Star™ (DE3) *E. coli* cells that express either Pepper-tagged 0× (0R-Pep) or 47×CAG-repeat RNA (47R-Pep). Scale bar, 5 μm. (**b**) Fluorescence imaging of BL21 Star™ (DE3) *E. coli* cells that express a pET-28c vector encoding Pepper-tagged near-infrared fluorescent protein (*Nir*-Pep) mRNA and a complementary strand-tagged 0R. Shown are the images before and after adding 1 μM HBC620 and 5 mM Mg^{2+} in DPBS buffer. Scale bar, 2 μm. (**c**) Average cellular Pepper and (**d**) NirFP fluorescence signals as measured in 366 and 512 individual cells were plotted before and after adding 1 μM HBC620 and 5 mM Mg^{2+}, respectively. Shown are violin plots with solid and dashed line indicating the median and interquartile value, respectively. The black cross indicates the mean value. Data was collected from at least three representative images. Two-tailed student's t-test: ***, $p<0.001$; ns, not significant, $p>0.05$.

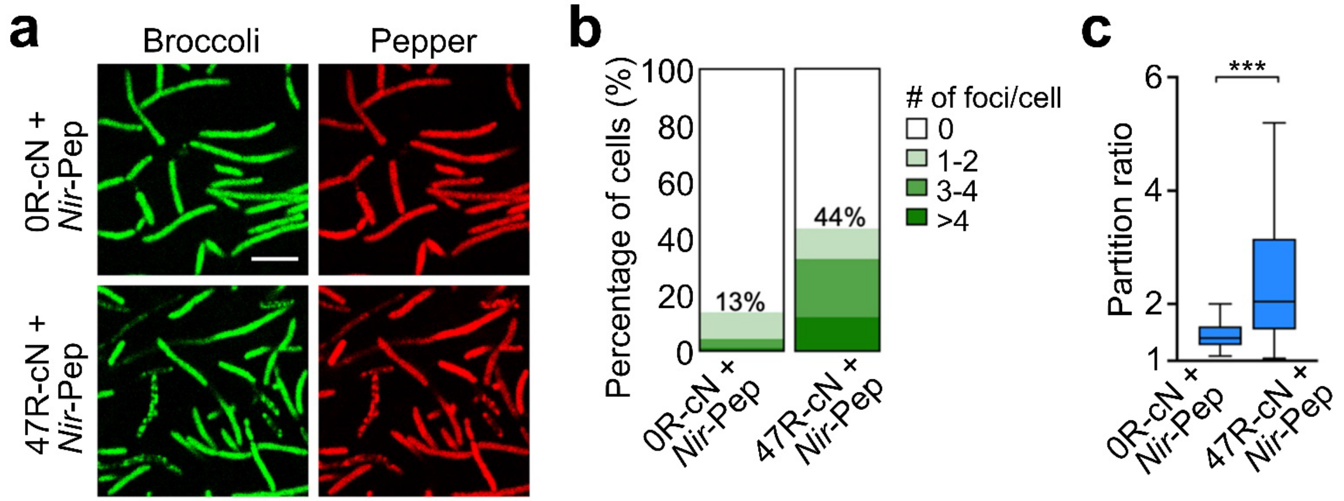

Figure S14. RNA condensate formation in living *P. aeruginosa* cells that carry an inducible, chromosomally integrated T7 RNA polymerase. (**a**) Fluorescence imaging of ADD1976 *P. aeruginosa* cells that express a pET-28c vector encoding Pepper-tagged near-infrared fluorescent protein RNA (*Nir*-Pep) and F30-2d×Broccoli-tagged 0× or 47×CAG repeats with a complementary strand (0R-cN or 47R-cN). Images were taken after adding 1 μM HBC620 and 200 μM DFHBI-1T in DPBS buffer. Scale bar, 5 μm. (**b**) Percentages of cells that contain different numbers of foci. Shown are 100% stacked columns of ADD1976 *P. aeruginosa* cells that express 0R-cN/*Nir*-Pep or 47R-cN/*Nir*-Pep. White, light green, green and dark green indicate the percentage of cells that exhibit 0, 1-2, 3-4 and >4 foci, respectively. ~13% of 0R-cN/*Nir*-Pep cells (95 in total) and 44% of 47R-cN/*Nir*-Pep cells (92 in total) contain at least one focus. (**c**) The partition ratio of each cellular foci as measured based on the Broccoli channel fluorescence levels. Shown are box plot with min-to-max whiskers. The top and bottom line, upper and lower box boundary, and inner line indicate the minimum and maximum data point excluding outliers, 75th, 25th percentile, and median of the data, respectively. Two-tailed student's t-test: ***, $p<0.001$.

Supplementary Table

Table S1. Sequences of RNAs used in this study. Sequences for the F30-2d×Broccoli, Pepper and near-infrared fluorescent protein (*NirFP*) are shown in green, red and magenta, respectively. The CAG or AC repeating sequences are bolded and the complementary sequences for trans-acting are underlined.

RNA name	Sequences (5'-3')
F30-2d× Broccoli	UUGCCAUGUGUAUGUGGGAGACGGUCGGGUCCAUCUGAGACGGUCGGGUCCAGAUAUUCGUAUC<br>UGUCGAGUAGAGUGUGGGCUCAGAUGUCGAGUAGAGUGUGGGCUCCCACAUACUCUGAUGAUCC<br>AGACGGUCGGGUCCAUCUGAGACGGUCGGGUCCAGAUAUUCGUAUCUGUCGAGUAGAGUGUGGG<br>CUCAGAUGUCGAGUAGAGUGUGGGCUGGAUCAUUCAUGGCAA
4R	F30-2d×Broccoli-UCUAGAGAAUUCGGUCUCA**CAGCAGCAGCAG**UUUUUUUCCGCGG
20R	F30-2d×Broccoli-UCUAGAGAAUUCGGUCUCA**CAGCAGCAGCAGCAGCAGCAGCAGCAGCAGCAGC**<br>**AGCAGCAGCAGCAGCAGCAGCAGCAG**UUUUUUUCCGCGG
31R	F30-2d×Broccoli-UCUAGAGAAUUCGGUCUCA**CAGCAGCAGCAGCAGCAGCAGCAGCAGCAGCAGC**<br>**AGCAGCAGCAGCAGCAGCAGCAGCAGCAGCAGCAGCAGCAGCAGCAGCAGCAGCAGCAG**UUUU<br>UUUCCGCGG
47R	F30-2d×Broccoli-UCUAGAGAAUUCGGUCUCA**CAGCAGCAGCAGCAGCAGCAGCAGCAGCAGCAGC**<br>**AGCAGCAGCAGCAGCAGCAGCAGCAGCAGCAGCAGCAGCAGCAGCAGCAGCAGCAGCAGCAGC**<br>**AGCAGCAGCAGCAGCAGCAGCAGCAGCAGCAGCAGCAGCAGCAG**UUUUUUUCCGCGG
70AC	F30-2d×Broccoli-UCUAGAGAAUUCGGUCUCA**ACACACACACACACACACACACACACACACACACA**<br>**CACACACACACACACACACACACACACACACACACACACACACACACACACACACACACACACAC**<br>**ACACACACACACACACACACACACACACACACACACACACA**UUUUUUUCCGCGG
Pepper	GGAUCCCCAAUCGUGGCGUGUCGGCCUGCUUCGGCAGGCACUGGCGCCGGGAUCC
Scrambled 0.1k RNA	CCUUUACCUGUAGGUGCGAUACCAUCGUAUCAUGUGAAGGGUACGUAGUUAAGAAAAUCACUAUG<br>UGCCCCGGCCUGUACGGUAAAACGGUAGGGUACGC
Scrambled 0.2k RNA	CUAGCAGGCGCGUAUCCAACCUACGCCACAAACUGGGCCGACGAGCAGGUGUUACAGGCCAGGAA<br>CAUAGGACUGUGUGCAGCAUCCUUGACUGAGGGAAGACUCGGCAAACUGUCCAUUCUCCGCAAGA<br>AGCAAUUGAAACCUUGCGACACAGUCAUGUUCUCGGUAGGAUCUACAUUGUACACUGAGAGCAGA<br>AAGCU
Scrambled 0.5k RNA	GAUGGCGGAUGUGUGACAUACACGACGCCAAAAGAUUUUGUUCCAGCUCCUGCCACCUCCGCUAC<br>GCGAGAGAUUAACCACCCACGAUGGCCGCCAAAGUGCAUGUUGAUAUUGAGGCUGACAGCCCAUU<br>CAUCAAGUCUUUGCAGAAGGCAUUUCCGUCGUUCGAGGUGGAGUCAUUGCAGGUCACACCAAAUG<br>ACCAUGCAAAUGCCAGAGCAUUUUCGCACCUGGCUACCAAAUUGAUCGAGCAGGAGACUGACAAA<br>GACACACUCAUCUUGGAUAUCGGCAGUGCGCCUUCCAGGAGAAUGAUGUCUACGCACAAAUACCA<br>CUGCGUAUGCCCUAUGCGCAGCGCAGAAGACCCCGAAAGGCUCGAUAGCUACGCAAAGAAACUGG<br>CAGCGGCCUCCGGGAAGGUGCUGGAUAGAGAGAUCGCAGGAAAAAUCACCGACCUGCAGACCGU<br>CAUGGCUACGCCAGACGCUGAAUCUCCUACCUUUUGCCUGCAUACA
Scrambled 1.0k RNA	CGUGACGUAUCACGCGGAGGGAUUCCUAGUGUGCAAGACCACAGACACUGUCAAAGGAGAAAGAG<br>UCUCAUUCCCUGUAUGCACCUACGUCCCCUCAACCAUCUGUGAUCAAAUGACUGGCAUACUAGCG<br>ACCGACGUCACACCGGAGGACGCACAGAAGUUGUUAGUGGGAUUGAAUCAGAGGAUAGUUGUGAA<br>CGGAAGAACACAGCGAAACACUAACACGAUGAAGAACUAUCUGCUUCCGAUUGUGGCCGUCGCAU<br>UUAGCAAGUGGGCGAGGGAAUACAAGGCAGACCUUGAUGAUGAAAAACCUCUGGGUGUCCGAGAG<br>AGGUCACUUACUUGCUGCUGCUUGUGGGCAUUUAAAACGAGGAAGAUGCACACCAUGUACAAGAA<br>ACCAGACACCCAGACAAUAGUGAAGGUGCCUUCAGAGUUUAACUCGUUCGUCAUCCCGAGCCUAU<br>GGUCUACAGGCCUCGCAAUCCCAGUCAGAUCACGCAUUAAGAUGCUUUUGGCCAAGAAGACCAAG<br>CGAGAGUUAAUACCUGUUCUCGACGCGUCGUCAGCCAGGGAUGCUGAACAAGAGGAGAAGGAGA<br>GGUUGGAGGCCGAGCUGACUAGAGAAGCCUUACCACCCCUCGUCCCCAUCGCGCCGGCGGAGAC

	GGGAGUCGUCGACGUCGACGUUGAAGAACUAGAGUAUCACGCAGGUGCAGGGGUCGUGGAAACA<br>CCUCGCAGCGCGUUGAAAGUCACCGCACAGCCGAACGACGUACUACUAGGAAAUUACGUAGUUCU<br>GUCCCCGCAGACCGUGCUCAAGAGCUCCAAGUUGGCCCCCGUGCACCCUCUAGCAGAGCAGGUG<br>AAAAUAAUAACACAUAACGGGAGGGCCGGCGGUUACCAGGUCGACGGAUAUGACGGCAGGGUCCU<br>ACUACCAUGUGGAUCGGCCAUUCCGGUCCCUGAGUUUCAAGCUUUGAGCGAGAGCGCCACUAUG<br>GUGUACAACGAAAGGGAGUUCGUCAACAGG
Scrambled<br>2.0k RNA	AAACUAUACCAUAUUGCCGUUCACGGACCGUCGCUGAACACCGACGAGGAGAACUACGAGAAAGU<br>CAGAGCUGAAAGAACUGACGCCGAGUACGUGUUCGACGUAGAUAAAAAAUGCUGCGUCAAGAGAG<br>AGGAAGCGUCGGGUUUGGUGUUGGUGGGAGAGCUAACCAACCCCCCGUUCCAUGAAUUCGCCUA<br>CGAAGGGCUGAAGAUCAGGCCGUCGGCACCAUAUAAGACUACAGUAGUAGGAGUCUUUGGGGUU<br>CCGGGAUCAGGCAAGUCUGCUAUUAUUAAGAGCCUCGUGACCAAACACGAUCUGGUCACCAGCGG<br>CAAGAAGGAGAACUGCCAGGAAAUAGUUAACGACGUGAAGAAGCACCGCGGGAAGGGGACAAGUA<br>GGGAAAACAGUGACUCCAUCCUGCUAAACGGGUGUCGUCGUGCCGUGGACAUCCUAUAUGUGGA<br>CGAGGCUUUCGCUUGCCAUUCCGGUACUCUGCUGGCCCUAAUUGCUCUUGUUAAACCUCGGAGC<br>AAAGUGGUGUUAUGCGGAGACCCCAAGCAAUGCGGAUUCUUCAAUAUGAUGCAGCUUAAGGUGAA<br>CUUCAACCACAACAUCUGCACUGAAGUAUGUCAUAAAAGUAUAUCCAGACGUUGCACGCGUCCAG<br>UCACGGCCAUCGUGUCUACGUUGCACUACGGAGGCAAGAUGCGCACGACCAACCCGUGCAACAAA<br>CCCAUAAUCAUAGACACCACAGGACAGACCAAGCCCAAGCCAGGAGACAUCGUGUUAACAUGCUUC<br>CGAGGCUGGGCAAAGCAGCUGCAGUUGGACUACCGUGGACACGAAGUCAUGACAGCAGCAGCAU<br>CUCAGGGCCUCACCCGCAAAGGGGUAUACGCCGUAAGGCAGAAGGUGAAUGAAAAUCCCUUGUAU<br>GCCCCUGCGUCGGAGCACGUGAAUGUACUGCUGACGCGCACUGAGGAUAGGCUGGUGUGGAAAA<br>CGCUGGCCGGCGAUCCCUGGAUUAAGGUCCUAUCAAACAUUCCACAGGGUAACUUUACGGCCACA<br>UUGGAAGAAUGGCAAGAAGAACACGACAAAAUAAUGAAGGUGAUUGAAGGACCGGCUGCGCCUGU<br>GGACGCGUUCCAGAACAAAGCGAACGUGUGUUGGGCGAAAAGCCUGGUGCCUGUCCUGGACACU<br>GCCGGAAUCAGAUUGACAGCAGAGGAGUGGAGCACCAUAAUUACAGCAUUUAAGGAGGACAGAGC<br>UUACUCUCCAGUGGUGGCCUUGAAUGAAAUUUGCACCAAGUACUAUGGAGUUGACCUGGACAGUG<br>GCCUGUUUUCUGCCCCGAAGGUGUCCCUGUAUUACGAGAACAACCACUGGGAUAACAGACCUGGU<br>GGAAGGAUGUAUGGAUUCAAUGCCGCAACAGCUGCCAGGCUGGAAGCUAGACAUACCUUCCUGAA<br>GGGGCAGUGGCAUACGGGCAAGCAGGCAGUUAUCGCAGAAAGAAAAAUCCAACCGCUUUCUGUGC<br>UGGACAAUGUAAUUCCUAUCAACCGCAGGCUGCCGCACGCCCUGGUGGCUGAGUACAAGACGGU<br>UAAAGGCAGUAGGGUUGAGUGGCUGGUCAAUAAAGUAAGAGGGUACCACGUCCUGCUGGUGAGU<br>GAGUACAACCUGGCUUUGCCUCGACGCAGGGUCACUUGGUUGUCACCGCUGAAUGUCACAGGCG<br>CCGAUAGGUGCUACGACCUAAGUUUAGGACUGCCGGCUGACGCCGGCAGGUUCGACUUGGUCUU<br>UGUGAACAUUCACACGGAAUUCAGAAUCCACCACUACCAGCAGUGUGUCGACCACGCCAUGAAGC<br>UGCAGAUGCUUGGGGGAGAUGCGCUACGACUGCUAAAACCCGGCGGCAUCUUGAUGAGAGCUUA<br>CGGAUACGCCGAUAAAAUCAGCGAAGCCGUUGUUUCCUCCUUAAGCAGAAAGUUCUCGUCUGCAA<br>GAGUGUUGCGCCCGGAUUGUGUCACCAGCAAUACAGAAGUGUUCUUGCUGUUCUCCAACUU
OxyS<br>(109 nt)	GAAACGGAGCGGCACCUCUUUUAACCCUUGAAGUCACUGCCCGUUUCGAGAGUUUCUCAACUCGA<br>AUAACUAAAGCCAACGUGAACUUUUGCGGAUCUCCAGGAUCCGC
lacY<br>(1254 nt)	AUGUACUAUUUAAAAAACACAAACUUUUGGAUGUUCGGUUUAUUCUUUUUCUUUUACUUUUUUAUC<br>AUGGGAGCCUACUUCCCGUUUUUCCCGAUUUGGCUACAUGACAUCAACCAUAUCAGCAAAAGUGA<br>UACGGGUAUUAUUUUUGCCGCUAUUUCUCUGUUCUCGCUAUUAUUCCAACCGCUGUUUGGUCUG<br>CUUUCUGACAAACUCGGGCUGCGCAAAUACCUGCUGUGGAUUAUUACCGGCAUGUUAGUGAUGUU<br>UGCGCCGUUCUUUAUUUUUAUCUUCGGGCCACUGUUACAAUACAACAUUUUAGUAGGAUCGAUUG<br>UUGGUGGUAUUUAUCUAGGCUUUUGUUUUAACGCCGGUGCGCCAGCAGUAGAGGCAUUUAUUGA<br>GAAAGUCAGCCGUCGCAGUAAUUUCGAAUUUGGUCGCGCGGAUGUUUGGCUGUGUUGGCUGG<br>GCGCUGUGUGCCUCGAUUGUCGGCAUCAUGUUCACCAUCAAUAAUCAGUUUGUUUUCUGGCUGG<br>GCUCUGGCUGUGCACUCAUCCUCGCCGUUUUACUCUUUUUCGCCAAAACGGAUGCGCCCUCUUC<br>UGCCACGGUUGCCAAUGCGGUAGGUGCCAACCAUUCGGCAUUUAGCCUUAAGCUGGCACUGGAA<br>CUGUUCAGACAGCCAAAACUGUGGUUUUUGUCACUGUAUGUUAUUGGCGUUUCCUGCACCUACGA<br>UGUUUUUGACCAACAGUUUGCUAAUUUCUUUACUUCGUUCUUUGCUACCGGUGAACAGGGUACGC<br>GGGUAUUUGGCUACGUAACGACAAUGGGCGAAUUACUUAACGCCUCGAUUAUGUUCUUUGCGCCA<br>CUGAUCAUUAAUCGCAUCGGUGGGAAAAACGCCCUGCUGCUGGCUGGCACUAUUAUGUCUGUAC

	GUAUUAUUGGCUCAUCGUUCGCCACCUCAGCGCUGGAAGUGGUUAUUCUGAAAACGCUGCAUAUG<br>UUUGAAGUACCGUUCCUGCUGGUGGGCUGCUUUAAAUAUAUUACCAGCCAGUUUGAAGUGCGUU<br>UUUCAGCGACGAUUUAUCUGGUCUGUUUCUGCUUCUUUAAGCAACUGGCGAUGAUUUUUAUGUCU<br>GUACUGGCGGGCAAUAUGUAUGAAAGCAUCGGUUUCCAGGGCGCUUAUCUGGUGCUGGGUCUGG<br>UGGCGCUGGGCUUCACCUUAAUUUCCGUGUUCACGCUUAGCGGCCCCGGCCCGCUUUCCCUGCU<br>GCGUCGUCAGGUGAAUGAAGUCGCUUAA
lacZ<br>(3048 nt)	GUCGUUUUACAACGUCGUGACUGGGAAAACCCUGGCGUUACCCAACUUAAUCGCCUUGCAGCACA<br>UCCCCCUUUCGCCAGCUGGCGUAAUAGCGAAGAGGCCCGCACCGAUCGCCCUUCCCAACAGUUG<br>CGCAGCCUGAAUGGCGAAUGGCGCUUUGCCUGGUUUCCGGCACCAGAAGCGGUGCCGGAAAGCU<br>GGCUGGAGUGCGAUCUUCCUGAGGCCGAUACUGUCGUCGUCCCCUCAAACUGGCAGAUGCACGG<br>UUACGAUGCGCCCAUCUACACCAACGUAACCUAUCCCAUUACGGUCAAUCCGCCGUUUGUUCCCA<br>CGGAGAAUCCGACGGGUUGUUACUCGCUCACAUUUAAUGUUGAUGAAAGCUGGCUACAGGAAGGC<br>CAGACGCGAAUUAUUUUUGAUGGCGUUAACUCGGCGUUUCAUCUGUGGUGCAACGGGCGCUGGG<br>UCGGUUACGGCCAGGACAGUCGUUUGCCGUCUGAAUUUGACCUGAGCGCAUUUUUACGCGCCGG<br>AGAAAACCGCCUCGCGGUGAUGGUGCUGCGUUGGAGUGACGGCAGUUAUCUGGAAGAUCAGGAU<br>AUGUGGCGGAUGAGCGGCAUUUUCCGUGACGUCUCGUUGCUGCAUAAACCGACUACACAAAUCAG<br>CGAUUUCCAUGUUGCCACUCGCUUUAAUGAUGAUUUCAGCCGCGCUGUACUGGAGGCUGAAGUU<br>CAGAUGUGCGGCGAGUUGCGUGACUACCUACGGGUAACAGUUUCUUUAUGGCAGGGUGAAACGC<br>AGGUCGCCAGCGGCACCGCGCCUUUCGGCGGUGAAAUUAUCGAUGAGCGUGGUGGUUAUGCCGA<br>UCGCGUCACACUACGUCUGAACGUCGAAAACCCGAAACUGUGGAGCGCCGAAAUCCCGAAUCUCU<br>AUCGUGCGGUGGUUGAACUGCACACCGCCGACGGCACGCUGAUUGAAGCAGAAGCCUGCGAUGU<br>CGGUUUCCGCGAGGUGCGGAUUGAAAAUGGUCUGCUGCUGCUGAACGGCAAGCCGUUGCUGAUU<br>CGAGGCGUUAACCGUCACGAGCAUCAUCCUCUGCAUGGUCAGGUCAUGGAUGAGCAGACGAUGG<br>UGCAGGAUAUCCUGCUGAUGAAGCAGAACAACUUUAACGCCGUGCGCUGUUCGCAUUAUCCGAAC<br>CAUCCGCUGUGGUACACGCUGUGCGACCGCUACGGCCUGUAUGUGGUGGAUGAAGCCAAUAUUG<br>AAACCCACGGCAUGGUGCCAAUGAAUCGUCUGACCGAUGAUCCGCGCUGGCUACCGGCGAUGAG<br>CGAACGCGUAACGCGAAUGGUGCAGCGCGAUCGUAAUCACCCGAGUGUGAUCAUCUGGUCGCUG<br>GGGAAUGAAUCAGGCCACGGCGCUAAUCACGACGCGCUGUAUCGCUGGAUCAAAUCUGUCGAUCC<br>UUCCCGCCCGGUGCAGUAUGAAGGCGGCGGAGCCGACACCACGGCCACCGAUAUUAUUUGCCCG<br>AUGUACGCGCGCGUGGAUGAAGACCAGCCCUUCCCGGCUGUGCCGAAAUGGUCCAUCAAAAAAUG<br>GCUUUCGCUACCUGGAGAGACGCGCCCGCUGAUCCUUUGCGAAUACGCCCACGCGAUGGGUAAC<br>AGUCUUGGCGGUUUCGCUAAAUACUGGCAGGCGUUUCGUCAGUAUCCCCGUUUACAGGGCGGCU<br>UCGUCUGGGACUGGGUGGAUCAGUCGCUGAUUAAAUAUGAUGAAAACGGCAACCCGUGGUCGGC<br>UUACGGCGGUGAUUUUGGCGAUACGCCGAACGAUCGCCAGUUCUGUAUGAACGGUCUGGUCUUU<br>GCCGACCGCACGCCGCAUCCAGCGCUGACGGAAGCAAAACACCAGCAGCAGUUUUUCCAGUUCCG<br>UUUAUCCGGGCAAACCAUCGAAGUGACCAGCGAAUACCUGUUCCGUCAUAGCGAUAACGAGCUCC<br>UGCACUGGAUGGUGGCGCUGGAUGGUAAGCCGCUGGCAAGCGGUGAAGUGCCUCUGGAUGUCG<br>CUCCACAAGGUAAACAGUUGAUUGAACUGCCUGAACUACCGCAGCCGGAGAGCGCCGGGCAACUC<br>UGGCUCACAGUACGCGUAGUGCAACCGAACGCGACCGCAUGGUCAGAAGCCGGGCACAUCAGCG<br>CCUGGCAGCAGUGGCGUCUGGCGGAAAACCUCAGUGUGACGCUCCCCGCCGCGUCCCACGCCAU<br>CCCGCAUCUGACCACCAGCGAAAUGGAUUUUUGCAUCGAGCUGGGUAAUAAGCGUUGGCAAUUUA<br>ACCGCCAGUCAGGCUUUCUUUCACAGAUGUGGAUUGGCGAUAAAAAACAACUGCUGACGCCGCUG<br>CGCGAUCAGUUCACCCGUGCACCGCUGGAUAACGACAUUGGCGUAAGUGAAGCGACCCGCAUUGA<br>CCCUAACGCCUGGGUCGAACGCUGGAAGGCGGCGGGCCAUUACCAGGCCGAAGCAGCGUUGUUG<br>CAGUGCACGGCAGAUACACUUGCUGAUGCGGUGCUGAUUACGACCGCUCACGCGUGGCAGCAUC<br>AGGGGAAAACCUUAUUUAUCAGCCGGAAAACCUACCGGAUUGAUGGUAGUGGUCAAAUGGCGAUU<br>ACCGUUGAUGUUGAAGUGGCGAGCGAUACACCGCAUCCGGCGCGGAUUGGCCUGAACUGCCAGC<br>UGGCGCAGGUAGCAGAGCGGGUAAACUGGCUCGGAUUAGGGCCGCAAGAAAACUAUCCCGACCG<br>CCUUACUGCCGCCUGUUUUGACCGCUGGGAUCUGCCAUUGUCAGACAUGUAUACCCCGUACGUC<br>UUCCCGAGCGAAAACGGUCUGCGCUGCGGGACGCGCGAAUUGAAUUAUGGCCCACACCAGUGGC<br>GCGGCGACUUCCAGUUCAACAUCAGCCGCUACAGUCAACAGCAACUGAUGGAAACCAGCCAUCGC<br>CAUCUGCUGCACGCGGAAGAAGGCACAUGGCUGAAUAUCGACGGUUUCCAUAUGGGGAUUGGUG<br>GCGACGACUCCUGGAGCCCGUCAGUAUCGGCGGAAUUCCAGCUGAGCGCCGGUCGCUACCAUUA<br>CCAGUUGGUCUGGUGUCAAAAAUAA

NirFP	CCAUCUUAGUAUAUUAGUUAAGUAUAAGAAGGAGAUAUACAUAUGGGAGAGGAUAGCGAGCUGAU<br>CUCCGAGAACAUGCACACGAAACUGUACAUGGAGGGCACCGUGAACGGCCACCACUUCAAGUGCA<br>CAUCCGAGGGCGAAGGCAAGCCCUACGAGGGCACCCAGACCUGUAAGAUCAAGGUGGUCGAGGG<br>CGGCCCUCUCCCCUUCGCCUUCGACAUCCUGGCUACCAGCUUCAUGUACGGCAGCAAAACCUUUA<br>UCAACCACACCCAGGGCAUCCCCGACUUCUUUAAGCAGUCCUUCCCUGAGGGCUUCACAUGGGAG<br>AGGAUCACCACAUACGAAGACGGGGGCGUGCUGACCGCUACCCAGGACACCAGCCUCCAGAACGG<br>CUGCCUCAUCUACAACGUCAAGAUCAACGGGGUGAACUUCCCAUCCAACGGCCCUGUGAUGCAGA<br>AGAAAACACUCGGCUGGGAGGCCAACACCGAGAUGCUGUACCCCGCUGACAGCGGUCUGAGAGG<br>CCAUAAUCAGAUGGCCCUGAAGCUCGUGGGCGGGGGCUACCUGCACUGCUCCCUCAAGACCACA<br>UACAGAUCCAAGAAACCCGCUAAGAACCUCAAGAUGCCCGGCUUCUACUUCGUGGACCGUAAACU<br>GGAAAGAAUCAAGGAGGCCGACAAAGAGACCUACGUCGAGCAGCACGAGAUGGCUGUGGCCAGGU<br>ACUGCGACCUGCCUAGCAAACUGGGGCACAGCUGA
Nir-Pep	Pepper-GUCGAC<u>GUUCACGUUGGCUUUAGUUAUUC</u>-*NirFP*
47R-cO	F30-2d×Broccoli-UCUAGAGAAUUCGGUCUCA**CAGCAGCAGCAGCAGCAGCAGCAGCAGCAGCAGC**<br>**AGCAGCAGCAGCAGCAGCAGCAGCAGCAGCAGCAGCAGCAGCAGCAGCAGCAGCAGCAGCAGC**<br>**AGCAGCAGCAGCAGCAGCAGCAGCAGCAGCAGCAGCAGCAGCAG**UUUUUUCCGCGGCUAGCA<br>UAACCCCUUG<u>GUUCACGUUGGCUUUAGUUAUUC</u>
47R-cY	F30-2d×Broccoli-UCUAGAGAAUUCGGUCUCA**CAGCAGCAGCAGCAGCAGCAGCAGCAGCAGCAGC**<br>**AGCAGCAGCAGCAGCAGCAGCAGCAGCAGCAGCAGCAGCAGCAGCAGCAGCAGCAGCAGCAGC**<br>**AGCAGCAGCAGCAGCAGCAGCAGCAGCAGCAGCAGCAGCAGCAG**UUUUUUCCGCGGCUAGCA<br>UAACCCCUUG<u>CGAAAAAGAGUAAAACGGCGAGGAUG</u>
47R-cN	F30-2d×Broccoli-UCUAGAGAAUUCGGUCUCA**CAGCAGCAGCAGCAGCAGCAGCAGCAGCAGCAGC**<br>**AGCAGCAGCAGCAGCAGCAGCAGCAGCAGCAGCAGCAGCAGCAGCAGCAGCAGCAGCAGCAGC**<br>**AGCAGCAGCAGCAGCAGCAGCAGCAGCAGCAGCAGCAGCAGCAG**UUUUUUCCGCGGACUAGU<br>AACUUGUUUAUUGCAGC<u>GAAUAACUAAAGCCAACGUGAAC</u>
47R-Pep	Pepper-GAUCAUUCAUGGCAAUCUAGAGAAUUCGGUCUCAC**CAGCAGCAGCAGCAGCAGCAGCAG**<br>**CAGCAGCAGCAGCAGCAGCAGCAGCAGCAGCAGCAGCAGCAGCAGCAGCAGCAGCAGCAGCAG**<br>**CAGCAGCAGCAGCAGCAGCAGCAGCAGCAGCAGCAGCAGCAGCAGCAGCAGCAG**UUUUUUCC<br>GCGG

References

1. Gordon,G.W., Berry,G., Liang,X.H., Levine,B., Herman,B. (1998) Quantitative fluorescence resonance energy transfer measurements using fluorescence microscopy. *Biophys. J.*, 74(5), 2702–2713.
2. Chen,H., Puhl,H.L., Koushik,S.V., Vogel,S.S., Ikeda,S.R. (2006) Measurement of FRET efficiency and ratio of donor to acceptor concentration in living cells. *Biophys. J.*, 91(5), 39–41.
3. Zal,T., Gascoigne,N.R. (2004) Photobleaching-corrected FRET efficiency imaging of live cells. *Biophys. J.,* 86(6), 3923–3939.
